# On quantitizing revisited

**DOI:** 10.3389/fpsyg.2024.1421525

**Published:** 2025-01-23

**Authors:** Anthony J. Onwuegbuzie

**Affiliations:** Faculty of Education, University of Johannesburg, Johannesburg, South Africa

**Keywords:** quantitizing, DIME-Driven Model of Quantitizing, mixed methods research, 1 + 1 = 1 integration approach, qualitative data, data transformation, inter-respondent matrix, quantitative analysis

## Abstract

This article builds on the highly cited 2009 article authored by Professor Emerita Margarete Sandelowski and her colleagues by critically reevaluating the process of quantitizing—transforming qualitative data into quantitative forms—a technique that has surprisingly not proliferated in academic research, presumably due to a shortage of methodological exploration in this area. This article responds to this shortfall by proposing a comprehensive meta-framework using the 5W1H approach, which outlines why, when, what, where, how, and who should engage in quantitizing, thereby integrating several frameworks and models across both mixed and multiple methods research. Central to this framework is the *DIME-Driven Model of Quantitizing*, which categorizes quantitizing into **D**escriptive, **I**nferential, **M**easurement, and **E**xploratory types, each enhancing the utility and precision of quantitizing. This innovative model supports the article's broader advocacy for quantitizing as a crucial methodological tool across diverse research traditions. This article explores the application and value of quantitizing across qualitative, quantitative, and mixed methods research traditions, demonstrating its broad relevance and transformative potential. It discusses the variable adoption of quantitizing based on differing philosophical perspectives related to ontology, epistemology, axiology, and methodology. Despite these differences, only a few research philosophies completely reject quantitizing. The article advocates for a balanced use of quantitizing to complement qualitative analyses and to enhance research clarity and applicability without compromising the richness of qualitative data. It serves as a comprehensive resource for understanding the complexities and utility of quantitizing, aiming to inspire researchers to consider this approach to enrich their analytical tools and to enhance the depth and applicability of their research findings.

## On quantitizing revisited

### A landmark publication on quantitizing

A decade and a half ago, the landmark mixed methodological article entitled, “On Quantitizing,” co-authored by Sandelowski et al. (2009), was published in the *Journal of Mixed Methods Research* (*JMMR*). Since its publication, it has received more than 1,000 citations. Their article, which is extremely thought-provoking, provides a comprehensive examination of quantitizing, covering philosophical and theoretical aspects. Sandelowski et al. (2009) define quantitizing as “the numerical translation, transformation, or conversion of qualitative data” (p. 208). More specifically, these authors describe quantitizing as representing

the process of assigning numerical (nominal or ordinal) values to data conceived as not numerical (or, following the previous discussion, to experience formed into words, visual displays, or something else conceived as qualitative). The not-numerical data typically referred to are segments of text in the form of written transcripts or field notes produced from interviews or participant observations that were themselves formed to accommodate the analyses planned (Emerson et al., 1995; Poland, 2002). The method used to accomplish this process is usually a variation of content, constant comparison, or domain analyses (e.g., Charmaz, 2006; Hsieh and Shannon, 2005; Spradley, 1979), whereby a priori and/or data-derived codes are attached to segments of text and numerical values are then assigned to those codes. (p. 2019–210)

Sandelowski et al. (2009) challenge readers to think critically about the practice of quantitizing in the field of mixed methods research. In particular, they critique the conventional practices and assumptions underlying quantitizing, providing a fresh perspective on how qualitative data are transformed into quantitative measures. Their discussion is relevant across various disciplines that utilize mixed methods research, making it a valuable resource for a broad audience of researchers.

Sandelowski et al. (2009) delve into the inherent challenges and subjective decisions involved in translating qualitative data into a numerical format that can be integrated with quantitative data. Further, they elaborate on the complexity involved in counting and making judgments about data. They emphasize that counting is not a straightforward, objective activity but is influenced by subjective judgments about what constitutes a countable object. That is, they illustrate how these activities are both inherently subjective and intersubjective. The subjectivity extends to the interpretation of qualitative data in research, wherein different analytical approaches can lead to different outcomes. Sandelowski et al. (2009) emphasize that the distinction between qualitative and quantitative data is not as rigid as traditionally thought, with each influencing and constituting the other. They discuss how the methodological choices, particularly in the design of data collection tools like questionnaires, inherently shape the data collected, affecting its interpretation and the validity of research conclusions. The theme of subjectivity is central, highlighting how qualitative data's transformation into numerical values is heavily dependent on human judgment and interpretation. The challenges of integrating qualitative and quantitative data are highlighted, particularly how qualitative data are forced to fit into pre-defined quantitative frameworks, which can distort the original data's meaning.

More specifically, Sandelowski et al. (2009) addressed the “foundational assumptions” (p. 208; e.g., qualitative and quantitative data are two types of data; quantitizing represents a unidirectional process that is generally different from qualitizing; counting is an unequivocal process), *judgments* (e.g., deciding on what and how to count), and *compromises* [e.g., “balancing numerical precision with narrative complexity” (p. 208)] involved in quantitizing. Further, they (p. 219–220) outlined the following three standpoints that illuminate the benefits of quantitizing: “conditional complementarity,” “critical remediation,” and “analytic alternation.” According to the authors, from the perspective of conditional complementarity, quantitizing is considered to enhance the value of qualitative data only if transforming it into quantitative form facilitates deeper insight and allows researchers to address significant questions or to test hypotheses in ways that would not be possible via other methods. Conversely, from the perspective of critical remediation, qualitative research enhances quantitative research by compensating for its deficiencies, namely, its failure to consider complexity, context, voice, and discourse. Here, qualitative research is viewed as crucial for ensuring the validity of quantitative methods. Consequently, the authors recognize the usefulness of redefining the concept of critical remediation to appreciate the value of quantitizing. In this context, refining the numerical precision of qualitative data and ensuring that their compatibility with quantitative data are seen as ways to augment the value of qualitative data.

Overall, Sandelowski et al. (2009) provide a detailed critique of the process of quantitizing, shedding light on its complexity and the nuanced decision-making involved. Further, they provide a deep and critical examination of the assumptions underlying the process of quantitizing in research. Their use of practical examples, such as different interpretations of questionnaire scales, is laudable, effectively illustrating the theoretical points and making complex concepts more accessible.

### A brief history of the word “quantitizing”

The term *quantitizing* dates back to at least 1894, as identified via an extensive search of the Google Scholar database. F. D. Allen first used the term in his discussion on ancient Roman poetry in an article wherein he critiqued the Word-accent Theory advocated by scholars like Keller, Thurneysen, and Westphal. Allen favored Alexander Reichardt's approach, which emphasized the quantitative analysis (i.e., quantitizing) of Saturnian meter, focusing on syllable length over stress patterns. He expressed this preference by noting, “Reichardt arrays himself with decision on the side of a *quantitizing* Saturnian, and dismisses the word-accent theory of Keller, Thurneysen and Westphal with brief comments that will seem wholly inadequate to the adherents of that doctrine” [emphasis added] (Allen, 1894, p. 207). This quotation highlights Allen's endorsement of Reichardt's methodology, which centers on the quantity of syllables as the primary structural element in verse, a common practice in classical metrics, particularly in Latin and Greek poetry. This traditional approach, known as quantitative meter, contrasts with the accentual meter, which bases the rhythmic structure on natural word stress. The debate over these approaches significantly has influenced the interpretation of ancient texts and the understanding of poetic forms in classical studies.

In terms of the Scopus database, the earliest documented use of the term *quantitizing* was that by Escudie et al. (1965). These authors described quantitizing as a method for converting a continuous range of values into a finite range of discrete values, a process essential in digital signal processing. This technique allows for the transformation of analog signals—continuous in nature—into digital signals, which are discrete and thus compatible with digital systems such as computers and digital circuits. In their discussion, the authors emphasized the increasing reliance on these methods, noting that signal processing “more and more often uses sampling and *quantitizing* methods” [emphasis added] (p. 161), underscoring the critical role these techniques play in the functionality of the circuits under study.

The next earliest article in the Scopus database is that by Schippers et al. (1967). These authors stated that “A device was designed which used gypsum and electrical conductivity means of detecting and *quantitizing* the amount of water emanating underground plant parts under natural conditions” [emphasis added] (p. 90). Here, the term “quantitizing” refers to the process of quantifying or measuring the amount of water emanating from underground plant parts in a precise, numerical form. More specifically, quantitizing involves turning a qualitative observation—water emanation—into quantifiable data that can be measured and analyzed statistically or numerically. The authors describe a device they designed that utilizes gypsum blocks and electrical conductivity as a means to detect and to measure (or to quantitize) the amount of water. Gypsum blocks are used in soil moisture measurement; they absorb water from the soil, which changes their electrical conductivity. This change in conductivity then can be measured and converted into an estimate of the soil moisture level. In this instance, the term “quantitizing” is used to describe the transformation of the qualitative aspect of moisture presence into quantitative, measurable units, thereby allowing for more precise scientific analysis and conclusions regarding the behavior and characteristics of water movement around underground plant parts. This quantification is essential for systematically studying environmental and biological processes, enabling the researchers to derive more detailed and accurate insights from their observations.

**Figure 1:**
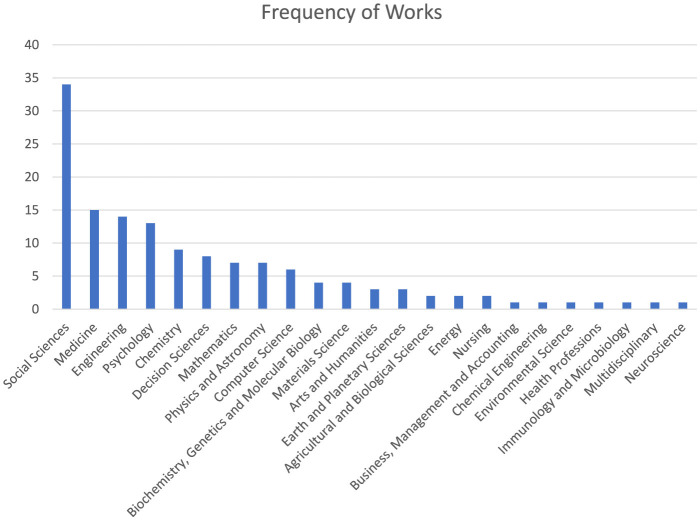
Frequency of scopus-indexed works across fields/disciplines that contain the word “quantitizing” or one of its variants in the title or abstract.

A systematic review of the Scopus database from 1960 to April 16, 2024, focused on the occurrences of the word “quantitizing” or one of its variants (i.e., *quantitize, quantitized, quantitizes, quantitising, quantitise, quantitised*, and *quantitises*) in titles or abstracts, reveals its application across at least 23 different fields and disciplines. Figure 1 illustrates the frequency of Scopus-indexed publications that mention “quantitizing” or its variants. From this figure, it is evident that the field of social sciences leads in the number of publications (*n* = 34), followed by medicine (*n* = 15), engineering (*n* = 14), and psychology (*n* = 13). This spread underscores the broad relevance and utility of the concept across a diverse range of scholarly areas.

In the social sciences, Caracelli and Greene (1993) are credited with initially discussing the concept of data transformation, specifically the conversion of one data type into another to facilitate combined analysis. However, it was Tashakkori and Teddlie (1998) who appear to have first used the term “quantitizing” or its variants within this discipline. In their seminal textbook on mixed methods research, they credited Miles and Huberman (1994) for inspiring their application of the technique. They specifically noted: “In this application, the qualitative data would be converted to numbers using the ‘quantitizing' technique described by Miles and Huberman (1994)” [emphasis in original] (p. 19).

Following the publication of Tashakkori and Teddlie's (1998) book, Sandelowski (2001) journal article appears to be the first to use the word “quantitizing”—doing so on one occasion when citing Tashakkori and Teddlie (1998). The term was first used in a Scopus-indexed article by Onwuegbuzie (2003). Intriguingly, Sandelowski et al. (2009) did not reference Tashakkori and Teddlie's (1998) work, but, instead, cited Sandelowski (2001) and Onwuegbuzie and Teddlie (2003) as the earliest sources employing the term *quantitizing*.

### Developments in quantitizing since 2009

**Figure 2:**
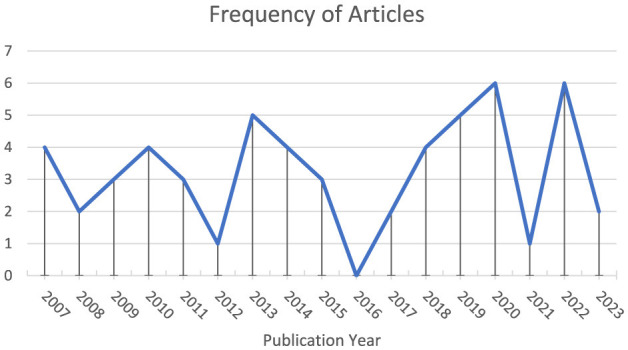
Frequency of articles published in the journal of mixed methods research that include a discussion of quantitizing.

Since Sandelowski et al.'s (2009) highly cited article, several works (e.g., Collingridge, 2013; Isaac et al., 2016; Kerrigan, 2014; Leal et al., 2018; Wao et al., 2011; Weaver-Hightower, 2014) have been published in which quantitizing has been explicitly demonstrated—as evidenced by the fact that the title includes the word *quantitizing* or one of its variants (i.e., *quantitize, quantitized, quantitizes, quantitising, quantitise, quantitised*, and *quantitises*).^1^ Interestingly, of the 346 articles (i.e., not including editorials, media reviews, notes, errata) published in *JMMR* at the time of writing (i.e., from Volume 1 and Issue 1 to Volume 18 and Issue 2), 55 have included the word *quantitizing* and/or one of its variants. This frequency indicates that 15.90% (i.e., 55/346) of all published *JMMR* articles to date have discussed/utilized quantitizing to some degree. Figure 2 displays the frequency of these *JMMR* articles by year. This figure indicates no linear trend (*p* = 0.83), no quadratic trend (*p* = 0.87), no cubic trend (*p* = 0.80), and no quartic trend (*p* = 0.72).^2^ Simply put, there is no clear publication pattern with regard to *JMMR* articles in which quantitizing has been used/discussed. Going beyond *JMMR*, an analysis of Scopus-indexed works published since 1960 also reveals no clear trend in works wherein quantitizing has been used/discussed.

A potential reason why the use of quantitizing has not increased over the years might stem from the lack of methodological articles published in this area. Moreover, with a few exceptions (e.g., Collingridge, 2013), methodological works devoted exclusively to the concept of quantitizing have tended largely to gravitate around its basic definition, as provided by Tashakkori and Teddlie (1998) and Sandelowski et al. (2009). Additionally, a small but significant body of work has presented arguments challenging the efficacy of quantitizing (e.g., Seltzer-Kelly et al., 2012) or presenting useful alternatives to quantitizing (e.g., Love and Corr, 2022), which might have played a role, however small, in further curtailing its widespread use. Another reason might stem from the justified concerns expressed about the process and outcomes associated with quantitizing, some of which were powerfully articulated by Sandelowski et al. (2009), and which will be presented in a subsequent section.

As such, the goal of the remainder of this editorial is to outline a meta-framework—comprising several frameworks and models, as well as both mixed methods research and multiple methods research approaches—for quantitizing qualitative data. As a starting point for our meta-framework, six important questions for information collection and problem solving should be answered—known as the Six *Ws* or 5W1H—namely, Why?, When?, What?, Where?, How?, and Who? The Six Ws/5W1H is a fundamental concept commonly used in several fields to obtain comprehensive information about a subject or an event. It refers to a set of questions whose answers are considered important in information-gathering or problem-solving. By addressing these six questions, one can form a comprehensive view of a situation, which is crucial for effective decision-making, problem-solving, and understanding. This meta-framework is extremely versatile and can be applied across various fields, from investigative reporting and business analysis to academic research. Each of these questions will be answered respectively in the following six major sections.

Importantly, Sandelowski et al. (2009) concluded their article with a call to action, stating: “This article is a beginning effort, and calls for more efforts, toward that end” (p. 220). Therefore, the following article, in part, is a response to their call by furthering the important dialogue that they initiated. It is designed to serve as a resource not only for mixed methods researchers, but also for quantitative researchers and qualitative researchers, because, as noted by Sandelowski et al. (2009), “Quantitizing is not confined to mixed methods research” (p. 210). The goal is to inspire these researchers to consider incorporating quantitizing into their data analysis strategies when appropriate. By doing so, this article hopes to broaden the analytical tools available to researchers, enhancing their ability to derive meaningful insights from their data.

### *Why* should researchers qualitize?

Quantitizing in mixed methods research serves multiple significant purposes, each enhancing the research process in distinct ways. First and foremost, quantitizing involves using both qualitative analyses (e.g., constant comparison of interview data to yield emergent themes) and, subsequently, quantitative analyses (e.g., descriptive statistics, such as frequencies, associated with these emergent themes) via the conduct of what Onwuegbuzie and Combs (2010) coined as crossover mixed analyses. Broadly defined, crossover mixed analysis refers to the method of applying an analytical technique traditionally used in one research tradition (e.g., quantitative) to data typically analyzed within another tradition (e.g., qualitative) (Combs and Onwuegbuzie, 2010; Greene, 2007; Onwuegbuzie and Combs, 2010; Onwuegbuzie et al., 2011; Onwuegbuzie, 2003; Onwuegbuzie and Teddlie, 2003). Crossover analysis can serve as the central analytic strategy in a study or as a component of various analytic steps. The extent to which crossover analysis is utilized should be guided by the specific research questions driving the study (Hitchcock and Onwuegbuzie, 2020). This technique facilitates a more integrative approach to data interpretation, allowing researchers to draw on the strengths of both quantitative and qualitative traditions to enhance the depth and breadth of their findings.

When optimally employed, crossover analysis in general and quantitizing in particular is consistent with Onwuegbuzie's (2017) 1 + 1 = 1 formula, which represents fuller integration of qualitative and quantitative research approaches (see also Onwuegbuzie and Hitchcock, 2019; Onwuegbuzie et al., 2018). Particularly, opting to quantitize during the conceptualization stage of the mixed methods research process might motivate the researcher(s) to collect, to analyze, and to interpret a diverse array of data types, thereby yielding full integration (Onwuegbuzie, 2022, 2023; Onwuegbuzie and Hitchcock, 2019, 2022). Creamer (2018) aptly notes, “in the highest-caliber mixed methods studies, the qualitative and quantitative strands are often so cleverly and iteratively interwoven that it becomes an exercise in semantics to disentangle the two” (p. 100). As will be discussed later, quantitizing under the 1 + 1 = 1 integration meta-framework not only enhances the depth of the research but also strengthens the coherence and richness of the results by intricately integrating different data types.

Second, quantitizing acts as a bridge, facilitating the integration and more comprehensive analysis of the qualitative and quantitative data (Onwuegbuzie, 2003; Onwuegbuzie and Teddlie, 2003). As such, this process allows for a fuller examination of all data, enhancing pattern recognition and the extraction of meaning (Miles and Huberman, 1994; Miles et al., 2014; Sandelowski et al., 2009). When systems thinking, achieved through quantitizing, is merged with pattern-matching from qualitative data analysis, it enables researchers to gain a deeper and more comprehensive understanding of a phenomenon (Cao, 2007). Furthermore, quantitizing can help to verify interpretations stemming from the qualitative data, ensuring a rigorous and transparent research process wherein the meaning-making process is clarified. Employing quantitizing at the conceptualization stage optimizes and clarifies analysis, enabling the recognition of complexities, contradictions, (ir)regularities, peculiarities, or idiosyncrasies in data that might otherwise remain obscured and uncommunicated (Morse and Niehaus, 2009).

Third, quantitizing can significantly contribute to the methodological robustness of a study. In particular, it can enable the empirical integration of qualitative data with quantitative datasets (Driscoll et al., 2007). This transformation is not just about juxtaposing different data types but about merging them to create a cohesive understanding (Driscoll et al., 2007). By converting qualitative data into a format amenable to statistical analysis, researchers can assimilate insights from diverse data collection methods, thereby enriching the analysis and strengthening the conclusions drawn. By employing quantitizing, researchers can attain a more detailed and nuanced comprehension of their data, as well as can help to clarify any confusion of meanings that might have stemmed from the qualitative data, thereby unlocking the potential for advanced statistical insights and more extensive generalizations. In addition, quantitizing can help qualitative findings to be placed in a more appropriate context.

Fourth, quantitizing can enhance the clarity and applicability of research findings. It translates complex qualitative insights into quantifiable data, making the findings more accessible and actionable for broader applications, including clinical and translational research efforts aimed at community betterment [Clinical and Translational Research Institute, 2024; Mace and Critchfield, 2010]. By facilitating new communication patterns and promoting behavioral changes, quantitizing can play a crucial role in translating research from basic discoveries (i.e., T1 research) to translating the research findings into daily practice (i.e., T2 research) to translating the research findings to the widespread community and clinical applications (i.e., T3 research; Abernethy and Wheeler, 2011; Ivankova et al., 2018; Woolf, 2008).

Fifth, quantitizing can enhance the empirical accuracy of the descriptive insights provided by qualitative approaches. It allows researchers to delve into minute details or to expand their focus to broader contexts (Willems and Raush, 1969). This increase in empirical precision is beneficial for improving the subsequent meta-inferences.

Sixth, quantitizing can foster innovation within the research process. Moreover, it can encourage researchers to approach data creatively, enhancing the interpretive richness and multivariate nature of the data (Onwuegbuzie and Dickinson, 2008; Tufte, 2006). This creative, critical, and reflexive engagement with data not only improves the depth of analysis but also increases the transparency of the findings. Furthermore, from a quantitative research perspective, quantitizing can act as a form of reliability check, such as by comparing the quantitized qualitative data with other quantitative data. In qualitative research terminology, this approach is considered a form of triangulation (Denzin, 1978) or, as Newman and Benz (1998) described, a method for assessing structural relationships.

In summary, as can be seen, there are several rationales for quantitizing. Indeed, this list of rationales is not exhaustive. However, hopefully, in this section, sufficient motivation has been provided for researchers at least to consider quantitizing when conducting mixed methods research studies. In essence, quantitizing is a multifaceted technique that extends beyond simple data conversion. It is a critical tool in the mixed methods research arsenal that enhances data integration, interpretation, transparency, and application, fostering a more nuanced and impactful understanding of research phenomena. This approach not only can help address the complexities inherent in diverse data types but also can enrich the dialogue between qualitative depths and quantitative precision.

### *When* should researchers quantitize?

Quantitizing is a strategic technique that researchers should consider utilizing when it has the potential, even if only partially, to address one or more of their research questions. The nature of the research questions themselves plays a crucial role in determining the suitability of quantitizing. For instance, using the typology conceptualized by Plano Clark and Badiee (2010), because quantitizing is applied to qualitative data, it is particularly relevant for *dependent research questions*—those that rely on outcomes derived from other questions—rather than for *independent research questions*, which relate to each other but do not depend on one another's outcomes. Furthermore, quantitizing techniques are applicable not only to *predetermined research questions* established at the onset of a study based on literature, practice, personal inclinations, or disciplinary considerations, but also to *emergent research questions*, which are new or modified research questions that develop during various phases of the research process (e.g., design, data collection, data analysis, or interpretation phases).

Quantitizing is especially pertinent to *general overarching mixed methods research questions*, which are broad inquiries addressed via both quantitative and qualitative research approaches (Plano Clark and Badiee, 2010). It is also useful for *hybrid mixed methods issue research questions*, which split a single research question into two distinct parts, each addressed by different research approaches (Plano Clark and Badiee, 2010). Additionally, quantitizing is crucial for *mixed methods procedural/mixing research questions*, which represent narrow questions that direct the integration of the qualitative and quantitative strands of the study. This is in contrast to *separate research questions*, wherein the quantitative and qualitative research questions remain distinct and unconnected.

The framework developed by Greene et al. (1989) offers a valuable way to conceptualize the goals of quantitizing, which can vary depending on whether the technique is applied in a single-stage or multiple-stage context. Single-stage quantitizing involves applying quantitizing techniques at one stage of the research process, whereas multiple-stage quantitizing entails the use of these techniques across two or more stages. In single-stage quantitizing, three of Greene et al.'s (1989) five goals are particularly relevant:

- *Complementarity*: Seeking to elaborate, to illustrate, to enhance, and to clarify findings from qualitative data with results from quantitized qualitative data, or vice versa.
- *Development*: Utilizing results from the quantitizing process to aid findings from qualitative data, enhancing the depth and understanding of those findings.
- *Expansion*: Broadening the scope and range of the study by employing multiple analytical strands, thereby enriching the study phases with findings from both qualitative data and quantitized results.

In multiple-stage quantitizing, the remaining goals of Greene et al. (1989) are critical:

- *Triangulation*: Comparing findings from quantitized data with results from other quantitative datasets to validate or to challenge the results.
- *Initiation*: Identifying paradoxes and contradictions that arise when comparing findings from quantitized data with quantitative results, potentially leading to a re-framing of the research questions.

In summary, quantitizing should be considered a versatile and integral part of mixed methods research, adaptable to various research questions and capable of fulfilling multiple research goals, thereby enriching the research process and outcomes.

### *When* should researchers *not* quantitize?

Although quantitizing is a valuable method in mixed methods research, there are specific contexts wherein it might be less beneficial or even counterproductive. It is crucial to emphasize that the purpose of this section is not to discourage the use of quantitizing entirely, but to provide clarity on situations wherein the technique might compromise rather than enhance the integrity of the data.

Researchers should avoid quantitizing when the intent is merely to transform qualitative data into quantitative form without regard for the richness and complexity of the original data. In such cases, quantitizing might lead to oversimplification and loss of depth in understanding the data's underlying narrative, particularly when there is no substantive alignment between the qualitative insights and the quantitative framework (Sandelowski et al., 2009). This can happen if the qualitative data are being manipulated or *forced* into numerical categories simply for the sake of comparison with existing quantitative datasets. This process might diminish the authenticity and richness of the qualitative findings, which are meant to capture nuances that numbers alone cannot convey.

Further, it is inappropriate to use quantitizing as a superficial method to compare qualitative findings to existing quantitative data, especially when there is no substantial justification for such comparison. In these cases, quantitizing inadvertently could undermine the qualitative analysis by imposing arbitrary or mismatched numerical structures on data that require a more nuanced interpretation.

However, this caution is by no means a blanket rejection of quantitizing. On the contrary, when used appropriately, quantitizing enhances mixed methods research by allowing qualitative insights to inform and to be informed by quantitative analysis, creating a more integrated and comprehensive approach. Researchers, especially beginning researchers (e.g., doctoral students), should approach quantitizing thoughtfully, ensuring that its application aligns with the research questions and the nature of the data, and should not shy away from using this powerful tool when it is methodologically justified. In essence, the 1 + 1 = 1 integration formula (Onwuegbuzie, 2017, 2022, 2023; Onwuegbuzie and Hitchcock, 2019, 2022) underscores the importance of integration rather than juxtaposition. Quantitizing should not be viewed as a unidirectional or mechanical process but rather as a dynamic, iterative one that enriches the research through the thoughtful blending of qualitative and quantitative insights.

### *What* should researchers quantitize

The technique of quantitizing entails transforming qualitative data into quantitative form (i.e., numerical codes) that can, in turn, be subjected to quantitative (i.e., statistical) analyses (Miles and Huberman, 1994; Sandelowski et al., 2009; Tashakkori and Teddlie, 1998). The sources of qualitative data suitable for conversion into numerical codes for quantitative analysis encompass the following four major groups as identified by Leech and Onwuegbuzie (2008): talk, observations, documents, and images (e.g., drawings, photographs, videos).^3^ Each of these sources provides a rich vein of qualitative data that, when quantitized, can be systematically analyzed to reveal patterns and insights not readily apparent through qualitative analysis alone. This approach allows researchers to bridge the interpretative depth of qualitative research with the statistical rigor of quantitative methodologies, enhancing the comprehensiveness and robustness of their findings.

The actual types of qualitative data that can be quantitized include codes, categories, sub-themes, themes, figures of speech, meta-themes, and narratives (i.e., prose or poetry). In the context of qualitative research, coding is a fundamental process wherein data elements—whether spoken words, phrases, or visual images—are encapsulated into succinct labels that capture their essence. A *code* can describe any unit of data, ranging from a brief utterance to an intricate image, across various media including still or moving visuals, drawings, photographs, or videos, presented in diverse sizes and durations. It represents the smallest point of meaning extracted from an object. Whether conveyed in physical or virtual spaces, codes distill the fundamental meaning from a data excerpt. Saldaña's (2021) identification of 33 coding strategies produces an array of code types, such as an *emotions code*, which discerns the underlying emotional states of participants.

When similar codes are aggregated, a *category* emerges, offering a broader interpretation of the data snippets. Moving up the abstraction ladder (Cartwright, 2012), a *subtheme* elevates the discussion by synthesizing these categories into a more cohesive narrative element, often encapsulated in a phrase or sentence. A *theme* further abstracts these ideas into significant elements or concepts that reveal patterned responses or meanings within the dataset. As such, it represents a higher level of abstraction or categorization than a subtheme. More specifically, it is an extended phrase or sentence that characterizes a major element, idea, or concept—representing some level of patterned response or meaning within the data set. Themes articulate major insights that are crucial for understanding the data's deeper layers.

Additionally, a *figure of speech* represents a specialized category of theme. These include a variety of rhetorical devices such as metaphors, which directly reference one concept by mentioning another; antitheses, which juxtapose functionally opposite ideas; hyperboles, which employ exaggeration for emphasis; analogies, which transfer meaning between subjects; metonymies, which involve the use of closely associated concepts interchangeably; puns, which play on multiple meanings of words; onomatopoeias, which mimic real sounds; and similes, which make direct comparisons. These devices can range from a single word to extended phrases, adding stylistic depth and enriching the narrative.

A *meta-theme* is an overarching phrase or sentence that encapsulates multiple themes, providing a higher order of interpretation and insight. Finally, in the context of research, particularly in social, behavioral, and health sciences, a *narrative* weaves together a logical and coherent story from selected codes, categories, subthemes, themes, and/or meta-themes. Although typically presented in prose, narratives also can be creatively expressed in poetic forms, offering a unique lens through which to view and to interpret the data.

It should be noted that the process of quantitizing qualitative data can differ based on the type of data being transformed. For instance, the prevalence of each code can be determined by counting their frequencies. In contrast, for categories, the frequencies of codes within each category can be calculated, and the categories can be analyzed by the proportion of codes they contain relative to the total number of codes. For sub-themes, the occurrences of each sub-theme within the data can be counted, and their distribution across different contexts or groups analyzed. Regarding themes, their presence across data sets can be measured, or a Likert-format scale can be used to rate their intensity or significance. The usage of each figure of speech can be counted and analyzed to understand their impact or frequency in the text. Meta-themes can be quantitized by counting how often they appear or are referenced across different data sets (e.g., focus group discussions, individual interviews), and their relative prominence can be compared. Narratives (i.e., prose or poetry) can be quantitized by breaking them down into codes, themes, or other units, and then analyzing the narrative length, complexity, and frequency of specific elements.

Overall, quantitizing these various forms of qualitative data can transform rich, descriptive information into a format amenable to statistical analysis. This transformation not only allows for a broader scale of analysis but also enhances the ability to communicate findings clearly and to apply them in practical settings, thereby bridging the gap between qualitative richness and quantitative rigor.

### *Where* should researchers quantitize?

Quantitizing can be effectively applied to qualitative data collected from both offline and online environments. Online environments encompass various forms of situated communication that occur through platforms like Facebook, X, Instagram, Snapchat, WhatsApp, Pinterest, WeChat, Reddit, blogs, wikis, forums, and listservs, as noted by Gerber et al. (2017). Moreover, in the era of big data, as discussed by Gerber et al. (in press), the potential to quantitize virtual data is growing at an unprecedented rate. This expanding scope reflects the increasing complexity and volume of digital interactions, providing rich qualitative datasets for quantitative analysis and enabling deeper insights into human behavior and societal trends.

The ability to transform intricate qualitative data sources into quantifiable information allows researchers to apply statistical tools and machine learning algorithms that can identify trends, predict outcomes, and model behaviors on a scale previously unimaginable. Such advancements significantly enhance the analytical power of researchers across disciplines, offering the means not only to track but also to anticipate changes in cultural patterns, social dynamics, and communication practices. As a result, quantitizing becomes a crucial technique in the toolkit of researchers, equipping them with the capabilities necessary to navigate and to interpret the vast landscapes of digital data.

### *How* should researchers quantitize?

**Figure 3:**
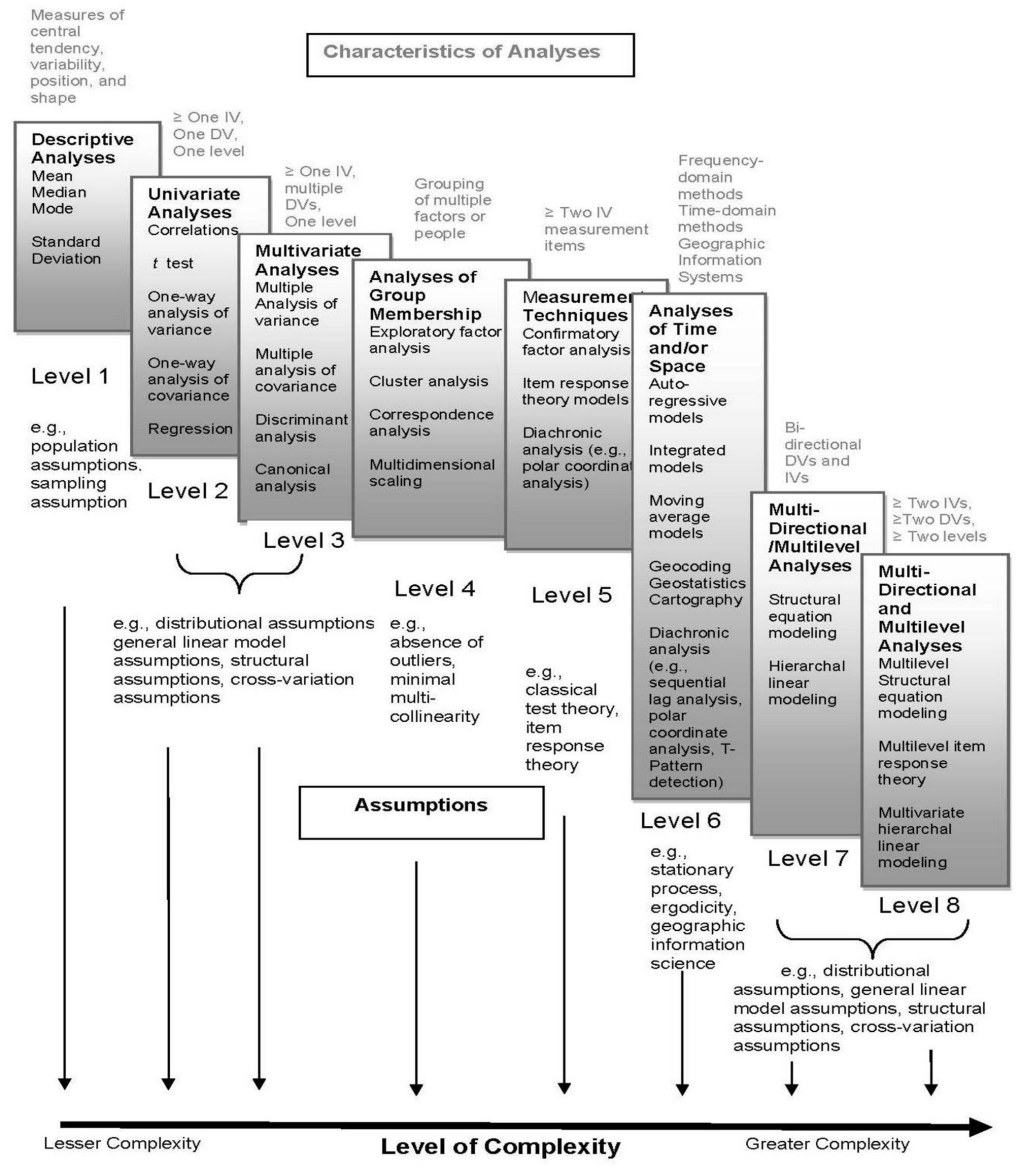
Quantitative analysis complexity continuum. Adapted from Ross and Onwuegbuzie (2014).

To date, in the context of mixed methods research, the process of quantitizing largely has remained elementary, as evidenced by Onwuegbuzie and Corrigan's (2018) meta-prevalence rate study, which involved identifying the prevalence of mixed methods research approaches across 46 prevalence rate studies between 1994 and 2015 that represented an array of fields and disciplines. Typically, this has involved straightforward enumerations—counting occurrences of codes, categories, sub-themes, themes, figures of speech, meta-themes, and narratives—often represented simply through frequency counts or employing basic descriptive statistics. This rudimentary approach to quantitizing aligns with the findings presented by Ross and Onwuegbuzie (2014). Their research reveals a consistent trend: when quantitative methods are integrated with qualitative research, there is a prevalent use of less complex analytical techniques. Specifically, within the framework of the eight distinct levels of quantitative analysis complexity illustrated in Figure 3, studies predominantly employ only the first three levels. These comprise descriptive analyses, univariate analyses, and, less frequently, multivariate analyses. This pattern underscores a cautious, perhaps conservative, utilization of quantitative complexity in mixed methods research, suggesting an opportunity for a deeper, more nuanced engagement with quantitative techniques to enrich qualitative findings. And a useful starting point for engaging in a wider variety of quantitative analyses is via the use of quantitizing.

Categorizing the types of quantitative analyses into eight levels of complexity, as shown in Figure 3, offers several benefits in the context of quantitizing qualitative data. First, it provides a clear and organized framework for researchers to understand and to select the appropriate quantitative analysis based on the complexity of their data and research questions. This helps in systematically planning and conducting analyses. Second, by categorizing the analyses, researchers, especially those who might not be as familiar with the array of quantitative analyses, can grasp more easily the progression from simple to complex analyses. This can facilitate learning and application of appropriate quantitizing techniques. Third, the continuum helps in matching the level of complexity of the analysis to the nature of the data. For instance, simpler descriptive statistics might be sufficient for basic quantitizing, whereas more complex multivariate analyses might be necessary for exploring relationships within and between multiple variables or groups. Fourth, different types of data (codes, categories, sub-themes, themes, figures of speech, meta-themes, and narratives) require different analytical approaches. The continuum ensures that researchers can choose methods that yield appropriate quantitization of each type of data, maintaining the integrity and richness of the original qualitative data. Fifth, using a standardized framework allows for consistency in data analysis across different studies. This can enhance the comparability of findings across various research studies and disciplines. Sixth, by progressing through levels of complexity, researchers can uncover deeper insights and more nuanced understandings of their data. For example, moving from simple frequency counts to multilevel structural equation modeling allows for a more sophisticated exploration of underlying patterns and relationships. Finally, documenting the analytical process using a complexity framework enhances the transparency and rigor of the quantitizing process. In turn, this can improve the credibility and reproducibility of the findings. In summary, the benefit of categorizing quantitative analyses into levels of complexity lies in providing a structured, understandable, and rigorous approach to quantitizing qualitative data, thereby enhancing the quality and clarity of research outcomes.

A valuable entry point for expanding the scope of quantitative analyses in mixed methods research is via the practice of quantitizing. By expanding the quantitizing process, researchers will be able to apply a broader array of sophisticated statistical techniques to qualitative information, thereby unlocking deeper insights and revealing patterns that might otherwise remain obscured. This approach not only will enrich the analytical depth of mixed methods research studies but also will enhance the rigor and generalizability of the findings, paving the way for a more integrated and comprehensive understanding of complex and complicated research phenomena.

To this end, in what follows, I present a four-level, meta-model of quantitizing that encapsulates a dynamic and intricate process far surpassing any existing conceptual frameworks. This meta-model delineates a stratified approach, meticulously structured to unfold across multiple levels. Each successive tier in this hierarchy represents an evolutionary step within the quantitizing process, guiding researchers through a systematic progression from foundational data categorization to complex analytical synthesis.

This meta-model represents a methodical and structured process, developed to illuminate the nuanced pathways by which qualitative data metamorphose into quantifiable insights. It is a paradigmatic blueprint, charting the course from raw narrative to numerical precision, and it promises to refine our understanding of the empirical world through the refined lens of quantitative clarity.

#### Level 1 quantitizing

Level 1 quantitizing, or first-level quantitizing, comprises the following four classes of quantitizing: **d**escriptive-based quantitizing, **i**nferential-based quantitizing, **m**easurement-based quantitizing, and **e**xploratory-based quantitizing. It is from the initials of these four foundational classes that the term *DIME-driven* quantitizing has been coined—elegant in its simplicity, yet profound in its significance. The DIME acronym serves not only as a mnemonic, but also as a metaphor for the value and precision inherent in this approach.

##### Descriptive-based quantitizing

Descriptive-based quantitizing involves converting qualitative data into quantitative metrics specifically to describe the characteristics of the data. This approach applies statistical techniques to summarize and to convey the patterns found in qualitative responses. Descriptive-based quantitizing includes the following four key groups of descriptive measures to outline these patterns effectively: measures of central tendency, measures of variation/dispersion, measures of position/relative standing, and measures of distributional shape. These measures are integral to the process of deriving meaning from data.

Measures of central tendency (e.g., mean, median, and mode) are used to describe the central point or typical value within a dataset. By quantitizing qualitative data to these metrics, researchers can determine the most typical or prevalent responses or themes within the data. Measures of variation/dispersion (e.g., range, interquartile range, standard deviation, and variance) help to describe how spread out the data points are within the dataset, giving insights into the consistency or diversity of qualitative responses. Measures of position/relative standing (e.g., percentiles, quartiles, *z*-scores, and *t*-scores) describe the position or ranking of individual data points within the overall data distribution of the qualitative responses. These measures help researchers understand how individual responses or themes compare to the overall group, highlighting outliers or particularly representative cases. Measures of distributional shape (e.g., skewness, kurtosis) provide insights into the symmetry and tail heaviness of the distribution of the qualitative data, important for understanding the underlying characteristics of the data.

In essence, descriptive-based quantitizing uses these statistical tools to provide a comprehensive statistical summary of qualitative data, making it possible to capture and to communicate the essence and nuances of the data in a structured, quantifiable form. This method is particularly useful in the initial stages of data analysis, wherein understanding the general trends and patterns is crucial.

The following examples of descriptive-based quantitizing illustrate how qualitative data can be transformed into quantitative metrics using various descriptive statistical measures, specifically, measures of central tendency, dispersion, position, and distributional shape. These examples span a variety of research contexts, demonstrating the flexibility of quantitizing in different fields and types of data collection, from interviews to focus groups. By applying these techniques, researchers can summarize qualitative data in a structured manner, revealing underlying patterns, trends, and distributions in the dataset. This allows for a deeper, more nuanced understanding of the data while preserving its richness.

###### Quantitizing qualitative data from survey data using frequency counts

The first example represents an article that ended up being the most downloaded article published in the *American Educational Research Journal* from 2007 to 2011. Onwuegbuzie et al. (2007) examined the 912 university students' perceptions of characteristics of effective college teachers who were administered a questionnaire asking them to identify and to rank between three and six characteristics that they believed effective college instructors should possess or demonstrate, as well as to provide a definition or description for each characteristic. A qualitative analysis, specifically constant comparison analysis, of these responses yielded nine themes, which are summarized by the acronym *RESPECTED*: ***R***esponsive, **E**nthusiast, **S**tudent-centered, **P**rofessional, **E**xpert, **C**onnector, **T**ransmitter, **E**thical, and **D**irector. These themes were quantitized as follows:

if a student listed a characteristic that was eventually unitized under a particular theme, then a score of 1 would be given to the theme for the student response; a score of 0 would be given otherwise. This dichomotization led to the formation of an interrespondent matrix (i.e., Student × Theme Matrix; Onwuegbuzie, 2003; Onwuegbuzie and Teddlie, 2003). Both matrices consisted only of 0 and 1 s. (p. 127)

This inter-respondent matrix then was subjected to descriptive-based quantitizing. In particular, “By calculating the frequency of each theme from the inter-respondent matrix, percentages were computed to determine the prevalence rate of each theme” (p. 127), which led to the numerous insightful findings, such as that student-centeredness was the most prevalent theme (i.e., 58.88% endorsement rate).

###### Quantitizing qualitative data from interviews using means as a measure of central tendency

For the second example, let us suppose that a researcher conducted a study examining the experiences of 100 first-time mothers. These mothers were interviewed about the challenges that they faced during their 1st year of motherhood. Using some form of qualitative analysis, their responses were categorized into the following five major themes: “sleep deprivation,” “emotional support,” “breastfeeding difficulties,” “time management,” and “partner involvement.” Each participant's response was scored for the presence (1) or absence (0) of each theme, yielding a binary inter-respondent matrix. The mean frequency of each theme then was calculated to identify the most commonly mentioned challenges. For example, if the “sleep deprivation” theme had a mean score of 0.85, this would indicate that 85% of the mothers discussed sleep deprivation as being a key challenge.

###### Using measures of variation/dispersion to quantitize focus group responses

For the third example, let us suppose that a researcher conducted a study of high school students' attitudes toward environmental conservation. Specifically, let us suppose that 10 focus groups were conducted that yielded a total of 80 students. After a thematic analysis, the students' responses were categorized into themes such as “recycling,” “energy conservation,” “climate change awareness,” and “sustainable practices.” These themes then were quantitized using measures of standard deviation and interquartile range to assess the variability of student engagement with each topic. The “recycling” theme had a standard deviation of 0.12 across focus groups, indicating consistent discussion across all groups, whereas “climate change awareness” had a higher standard deviation of 0.45, reflecting a more varied emphasis across groups.

###### Quantitizing open-ended survey responses using measures of position/relative standing

For the fourth example, let us suppose that a researcher conducted a survey of employee satisfaction in a multinational company. Let us suppose further that open-ended responses from 500 employees about their job satisfaction were coded into five major themes: “work-life balance,” “career development opportunities,” “management support,” “salary satisfaction,” and “team collaboration.” Each response was assigned a score based on the presence or absence of these themes. To analyze further these responses, the data were converted into percentiles. For example, the “work-life balance” theme ranked in the 80th percentile, meaning that 80% of respondents mentioned it as a key factor in their job satisfaction, whereas “team collaboration” ranked in the 30th percentile, suggesting that it was less frequently mentioned.

###### Applying measures of distributional shape to quantitize narrative data

For the fifth example, let us suppose that a researcher conducted a study investigating patient experiences with chronic illness that involved analyzing narratives from 200 patients. These narratives were coded into four themes: “treatment satisfaction,” “doctor-patient communication,” “access to care,” and “emotional resilience.” Using measures of skewness and kurtosis, researchers analyzed the distribution of these themes. The “treatment satisfaction” theme showed a positive skewness of 1.5, indicating that most patients had positive responses, but a few reported extreme dissatisfaction—using Westfall and Henning's (2013) criteria, wherein skewness coefficients between 1 and 2 suggest moderate asymmetry, whereas higher values represent more extreme asymmetry. The “doctor-patient communication” theme had a kurtosis of 2.3, suggesting that most patients had moderately similar experiences, with few outliers reporting exceptionally good or bad communication experiences—using Westfall and Henning's (2013) criteria, wherein kurtosis coefficients close to 3 indicate normal distribution, with lower values (< 3) suggesting a flatter (i.e., platykurtic) distribution and higher values (>3) indicating a distribution with more outliers or extreme values (i.e., leptokurtic distribution).

##### Inferential-based quantitizing

Inferential-based quantitizing involves transforming qualitative data into estimations or predictions via methods such as general linear model (GLM) analysis—for example, the correlation coefficient, independent samples *t*-test, analysis of variance (ANOVA), multiple analysis of variance (MANOVA), canonical correlation analysis, structural equation modeling (SEM), and hierarchical linear modeling (HLM)—tailoring the data for more predictive utility. The purpose of inferential-based quantitizing is to make inferences about a larger population from a sample. This approach enables researchers to extend conclusions beyond the immediate data set to broader contexts, which can be essential in validating hypotheses and supporting generalizations. For instance, in educational research, inferential-based quantitizing might involve coding and quantitizing qualitative responses from a focus group of teachers discussing educational technologies. The quantitized data then could be used to perform a logistic regression to predict the likelihood of technology adoption based on factors such as age, experience, and attitudes. The results could be generalized to suggest broader trends across schools or regions, providing that the sample is representative.

The following examples illustrate how inferential-based quantitizing enables researchers to transform qualitative insights into quantitative data that can be analyzed using various inferential techniques. Through methods such as canonical correlation analysis, multiple regression, ANOVA, logistic regression, and structural equation modeling, researchers can explore deeper relationships and test hypotheses across larger populations. By doing so, researchers can make predictions, examine relationships, and draw conclusions that extend beyond the immediate sample, thereby enhancing the generalizability and predictive power of their findings. This approach allows for a more comprehensive understanding of complex phenomena while preserving the richness of the original qualitative data.

###### Using canonical correlation analysis to examine the relationship between demographic variables and qualitatively derived themes

Circling back to the Onwuegbuzie et al.'s (2007) investigation, these researchers conducted a canonical correlation analysis in order to examine the relationship between the nine quantitized themes and the eight demographic variables (i.e., gender, race, level of student, preservice teacher status, age, GPA, number of credit hours taken, and number of offspring). Among the numerous results stemming from this analysis was the finding that

gender, race, age, level of student, preservice teacher status, number of offspring, and number of credit hours are related in some combination to enthusiast, student centered, professional, ethical, expert, and director. Of the demographic variable set, only GPA did not appear to play a role in the prediction of the themes. (p. 140)

In essence, inferential-based quantitizing transforms qualitative insights into quantitative data that can be analyzed statistically to draw conclusions about larger populations, providing a powerful tool for substantiating research findings in mixed methods research studies.

###### Using multiple regression to predict employee job satisfaction based on qualitatively derived themes

For the second example, let us suppose that researchers conducted a study exploring the job satisfaction of employees in a multinational company. These researchers conducted semi-structured interviews with 200 employees. Qualitative responses were coded into themes such as “work-life balance,” “career development,” “management support,” and “work environment.” These themes then were quantitized, and a multiple regression analysis was performed to predict overall job satisfaction. The researchers used the presence of these quantitized themes as independent variables to predict the dependent variable of job satisfaction scores from a quantitative survey. Results indicated that “management support” and “career development” were the strongest predictors of job satisfaction, accounting for 40% of the variance in the satisfaction scores. This finding would suggest that enhancing management support and career development programs could improve job satisfaction across the organization.

###### Performing ANOVA to compare student engagement across different learning styles

For the third example, let us suppose that researchers conducted an educational study wherein they interviewed 150 high school students about their engagement levels in different learning environments (e.g., group work, individual projects, and technology-assisted learning). The qualitative responses were coded and quantitized, categorizing engagement into three themes: “high engagement,” “moderate engagement,” and “low engagement.” Researchers then performed a one-way ANOVA to compare engagement levels across three different learning styles: visual, auditory, and kinesthetic. Let us suppose further that the analysis revealed a statistically significant difference in engagement levels, with visual learners reporting the highest engagement in technology-assisted learning [*F*_(2, 147)_ = 6.32, *p* < 0.01] that represented a large effect size (η^2^ = 0.14). This inferential analysis would allow the researchers to conclude that, for these high school students, technology-assisted learning is particularly effective for visual learners.

###### Logistic regression to predict health behaviors from qualitatively coded themes

For the fourth example, let us suppose that researchers undertook a health behavior study wherein they conducted focus groups with 120 participants discussing their experiences with adopting healthy habits. The qualitative responses were coded into themes such as “exercise frequency,” “dietary choices,” “emotional support,” and “time management.” These themes then were quantitized, and a logistic regression was conducted to predict whether participants adopted a healthy lifestyle (binary outcome: 0 = no, 1 = yes) based on the coded themes. Let us suppose further that the logistic regression model showed that “exercise frequency” and “emotional support” were statistically significant predictors of adopting a healthy lifestyle (*p* < 0.05), whereas “time management” was not a statistically significant predictor. This inferential-based quantitizing would allow the researchers to predict the likelihood of a healthy lifestyle being adopted based on the presence of certain qualitative factors in participants' narratives.

###### Structural equation modeling to analyze the relationships between workplace stressors and burnout

For the fifth example, let us suppose that researchers conducted in-depth interviews with 100 nurses to explore the sources of workplace stress and how these stressors contributed to burnout. Qualitative data were coded into stressor categories such as “workload,” “emotional strain,” “interpersonal conflict,” and “lack of autonomy.” These categories then were quantitized and used in a structural equation modeling (SEM) analysis to examine the relationships between these workplace stressors and burnout levels (measured by a standardized burnout scale). Let us suppose further that the SEM analysis revealed that “emotional strain” had the strongest direct effect on burnout (β = 0.65, *p* < 0.001), whereas “lack of autonomy” had an indirect effect, mediated by “workload.” This inferential approach would allow the researchers to generalize the relationships between stressors and burnout to the broader nursing population, providing actionable insights for healthcare administrators.

##### Measurement-based quantitizing

Measurement-based quantitizing involves converting qualitative data into quantitative data specifically for the purposes of measurement, including the development of instruments and score-validation of constructs. This approach typically involves the systematic transformation of qualitative observations into quantifiable scales or metrics that can be empirically tested and score-validated. It allows researchers to measure complex constructs that are not directly observable but can be inferred from qualitative data. Techniques such as Rasch modeling and item response theory (IRT) are commonly used in this process. These techniques help in assessing the properties of the measurement tools, including their score reliability (consistency of the scores yielded by the instruments) and item functioning (how individual items contribute to the overall measurement), ensuring that the instruments and constructs are robust and accurately reflect the variables they measure.

Consider a study aimed at understanding the barriers to exercise among middle-aged adults. Researchers might initially conduct qualitative interviews to gather detailed descriptions of perceived barriers. Using measurement-based quantitizing, these barriers could be categorized and transformed into a survey instrument with scaled items measuring each barrier's perceived impact. The survey then could be administered to a larger population to score-validate the scale and quantitatively assess the prevalence and impact of each barrier, ultimately contributing to targeted interventions.

Returning to the Onwuegbuzie et al. (2007) study, although the authors did not do this, based on their large sample size (i.e., *n* = 912), they could have conducted a Rasch analysis to identify the themes' prevalence or rarity across responses, thereby revealing their relative difficulty or ubiquity. Alternatively, they could have conducted a 2-parameter IRT model to identify not only the themes' prevalence, but also their efficacy in differentiating among participants' qualitative responses.

The following additional examples demonstrate how measurement-based quantitizing, using techniques such as Rasch modeling and various forms of IRT, transforms qualitative data into scales that can be empirically tested. These techniques particularly are useful in developing and score-validating instruments that measure complex constructs derived from qualitative insights. By applying these models, researchers can assess the difficulty, discrimination, guessing, and carelessness parameters of the measurement tools, ensuring that the constructs being measured are reliable, valid, and reflective of the complexities of the qualitative data. This process allows for a more precise and robust measurement of phenomena that are not directly observable but can be inferred through systematic quantitizing.

###### Rasch modeling to measure teaching effectiveness

For the first example, let us suppose that researchers undertook a study on teaching effectiveness. They conducted qualitative interviews with 500 students who described the traits of highly effective teachers. The responses were coded into themes such as “classroom engagement,” “clarity of instruction,” and “empathy.” Using Rasch modeling, these themes were transformed into a scale with items measuring each trait. Rasch analysis was applied to assess the scalability and difficulty of each item, identifying which traits were consistently rated as being more challenging for teachers to achieve. Let us suppose further that the results indicated that “classroom engagement” had a higher item difficulty compared to “empathy,” meaning that it was less frequently observed by students but highly valued when present. Rasch modeling would allow the researchers to create a linear scale to assess teaching effectiveness based on student perceptions.

###### Two-parameter IRT to measure resilience in healthcare workers

For the second example, let us suppose that researchers conducted focus groups with healthcare workers to explore the challenges that they face in maintaining resilience during crises. The qualitative data revealed several key factors, including “emotional support,” “work-life balance,” “personal coping strategies,” and “institutional support.#” These factors were transformed into items for a resilience scale. Using two-parameter IRT, researchers quantified both the difficulty (how challenging it was for healthcare workers to embody each factor) and discrimination (how well each item distinguished between high and low resilience levels). Let us suppose further that the analysis demonstrated that “institutional support” had a high discrimination parameter, meaning that it was a strong predictor of resilience, whereas “emotional support” had a lower difficulty parameter, indicating that it was a commonly available resource. This would allow the development of a refined instrument to measure resilience in healthcare settings.

###### Three-parameter IRT to measure public perceptions of climate change

For the third example, let us suppose that researchers undertook a study on public perceptions of climate change. They conducted interviews with 1,000 participants to understand the various factors shaping their views. The interviews were coded into themes such as “scientific trust,” “personal responsibility,” “economic impact,” and “government action.” These themes were quantitized and used to develop a survey measuring public concern over climate change. Using three-parameter IRT, the researchers assessed difficulty, discrimination, and guessing (the likelihood that respondents with low knowledge might guess a correct answer). Let us suppose further that the results indicated that “scientific trust” was highly discriminative of climate change concern, with a moderate difficulty level, but “government action” had a higher guessing parameter, suggesting that some respondents might not fully understand the item but answered in alignment with social expectations. This model would help to refine the measurement of public perception by addressing potential guessing biases.

###### Four-parameter IRT to measure ethical decision-making among professionals

For the fourth example, let us suppose that researchers undertook a study investigating ethical decision-making among professionals. These researchers interviewed 300 participants from various industries. The interviews were coded into themes like “personal integrity,” “corporate policies,” “peer influence,” and “legal considerations.” These themes then were quantitized and used to create a scale measuring ethical decision-making. Using four-parameter IRT, the researchers evaluated difficulty, discrimination, guessing, and carelessness (the probability that respondents might not be paying full attention when answering). Let us suppose further that the analysis revealed that “personal integrity” had a high difficulty and discrimination value, meaning that it was challenging but crucial for ethical decision-making. However, the “corporate policies” theme showed a higher carelessness parameter, suggesting that some participants might not take corporate policies into account when making ethical decisions. This would allow the researchers to refine their instrument by accounting for potential carelessness in responses.

Overall, measurement-based quantitizing is a critical bridge between qualitative explorations of complex constructs and their rigorous, systematic quantification, enabling deeper insights and broader applications in scientific research. This methodological approach not only enriches the precision and interpretability of qualitative data, but also extends the reach of these findings into domains traditionally dominated by quantitative analysis. By transforming nuanced, subjective responses into quantifiable metrics, researchers more effectively can compare, replicate, and build on their findings, ultimately contributing to the advancement of knowledge across diverse disciplines.

##### Exploratory-based quantitizing

Exploratory-based quantitizing refers to the process of transforming qualitative data into quantitative data for the purpose of exploring new insights, uncovering underlying patterns, discerning group memberships, and identifying potential relationships within the qualitative data. This is achieved through methodologies like exploratory factor analysis, cluster analysis, or correspondence analysis, each aiming to uncover underlying patterns or structures within the data. This approach is particularly useful in the early stages of research when hypotheses are not yet clearly defined, and the goal is to generate new hypotheses and theories based on the data. In a social science study, researchers might conduct detailed interviews to collect qualitative data on community attitudes toward a new public policy. Through exploratory-based quantitizing, the researchers could code responses into quantitative data and apply cluster analysis to identify distinct groups within the community based on their attitudes. This could reveal unexpected patterns, such as specific demographic groups that are particularly supportive or critical of the policy. These findings then could be used to refine further studies or to develop targeted communication strategies.

The following additional examples illustrate how exploratory-based quantitizing transforms qualitative data into quantifiable metrics, enabling the use of exploratory techniques like principal components analysis (PCA), exploratory factor analysis (EFA), cluster analysis, multidimensional scaling (MDS), and correspondence analysis to uncover patterns, relationships, and groupings within the data. These methods allow researchers to delve deeper into the qualitative responses, revealing hidden structures and connections that might not be immediately apparent. By applying these techniques, researchers can generate new insights and hypotheses, laying the foundation for further research and theory development in a variety of fields, from consumer behavior to education and mental health. This process helps to bridge the gap between exploratory qualitative work and the more structured, data-driven approach of quantitative research.

###### Principal components analysis to identify the hierarchical structure of themes pertaining to characteristics of effective college teachers

For the first example, returning to the study by Onwuegbuzie et al. (2007), the researchers conducted a PCA on the nine RESPECTED themes, after transforming the zero-order correlation coefficients to tetrachoric correlation coefficients. This step was crucial to tailor the PCA to the specific characteristics of the binary data (i.e., “1” vs. “0”). The analysis identified four meta-themes—**c**ommunicator (43.7% of characteristics per meta-theme), **a**dvocate (81.0%), **r**esponsible (41.1%), and **e**mpowering (59.6%)—which were collectively referred to by the acronym CARE. This CARE model, when combined with the nine RESPECTED themes, led to the development of the CARE-RESPECTED Model of Effective College Teaching.

###### Exploratory factor analysis to identify dimensions of consumer preferences

For the second example, let us suppose that researchers undertook a study examining consumer preferences for sustainable products. They conducted in-depth interviews with 300 participants to explore their attitudes toward eco-friendly packaging, ethical sourcing, and product durability. The qualitative responses were coded into themes and transformed into quantitative data. Using EFA, the researchers sought to identify the underlying dimensions of consumer preferences. Let us suppose further that the EFA revealed three key factors: “Environmental Responsibility,” “Product Quality,” and “Brand Trust,” which explained 65% of the variance in the responses. These factors then could be used to develop a comprehensive framework for understanding consumer motivations related to sustainability, guiding further studies and product development.

###### Cluster analysis to group participants by mental health coping strategies

For the third example, let us suppose that researchers undertook a study focused on mental health. These researchers conducted semi-structured interviews with 500 participants discussing how they cope with anxiety and depression. The interviews were qualitatively analyzed and coded into specific coping strategies, such as “exercise,” “meditation,” “social support,” and “self-care routines.” After quantitizing the data, the researchers applied cluster analysis to group participants based on their use of these strategies. Let us suppose further that the analysis revealed three distinct clusters: “Holistic Copers,” who emphasized meditation and self-care, “Active Copers,” who relied on physical activity and social support, and “Passive Copers,” who primarily used minimal strategies. These insights would help researchers better understand coping behavior patterns and tailor future mental health interventions to the specific needs of each group.

###### Multidimensional scaling to explore perceptions of workplace diversity

For the fourth example, let us suppose that researchers undertook a study wherein they interviewed 200 employees from a variety of industries to investigate their perceptions of workplace diversity initiatives. The qualitative responses were coded into themes, such as “inclusivity,” “equal opportunity,” “cultural representation,” and “workplace harmony.” After transforming these themes into quantitative data, the researchers applied MDS to map the relationships among the different perceptions. Let us suppose further that the MDS plot revealed that “inclusivity” and “equal opportunity” were perceived as being closely related, whereas “cultural representation” and “workplace harmony” formed another distinct cluster. This exploration would help researchers identify the nuanced ways that employees viewed diversity efforts, guiding further inquiry into specific areas for organizational improvement.

###### Correspondence analysis to uncover relationships in educational research

For the fifth example, let us suppose that researchers undertook a study investigating the teaching styles of high school educators. These researchers conducted open-ended interviews with 100 teachers to understand their instructional approaches. The qualitative data were coded into categories, such as “student-centered teaching,” “lecture-based instruction,” and “technology integration.” These categories were quantitized, and correspondence analysis was conducted to examine the relationships between teaching styles and demographic variables, such as years of experience and subject area. Let us suppose further that this analysis revealed that teachers with more than 10 years of experience were more likely to use “lecture-based instruction,” whereas younger teachers tended to favor “technology integration.” Correspondence analysis would uncover these associations, providing valuable insights into how teaching practices evolve over time and across subject areas.

Thus, exploratory-based quantitizing is a powerful tool in mixed methods research, enabling researchers to delve into qualitative data with quantitative tools to uncover new patterns and relationships that can guide future research directions and decision-making. This integrative approach not only amplifies the analytical capabilities of researchers, but also fosters a more comprehensive understanding of data by bridging the methodological divide. With exploratory quantitizing, subtle nuances and complex dynamics within the data are revealed, offering a richer, more detailed landscape for academic inquiry and practical application.

#### Level 2 quantitizing

Level 2 quantitizing, or second-level quantitizing, comprises spatial-based quantitizing and time-based quantitizing. Each of these types of second-level quantitizing will be described in the following sections.

##### Spatial-based quantitizing

Spatial-based quantitizing refers to the process of transforming qualitative data into quantitative forms that specifically include spatial (geographical) components. This approach can be used in studies wherein the spatial distribution of the phenomenon of interest is significant and can help in understanding patterns, relationships, or trends that are influenced by geographical variables. For instance, spatial-based quantitizing can be achieved by quantitizing qualitative data based on their geographical attributes, such as proximity, area, density, or distribution across a map. Spatial-based quantitizing can involve the use of tools like Geographic Information Systems (GIS) to visualize the quantitized qualitative data on maps. This would allow researchers to see patterns that might not be apparent from the raw qualitative data alone, such as clustering of phenomena, variation across different regions, or the influence of geographical features on social or environmental factors. Techniques such as spatial analysis, clustering, or heat mapping can be used to interpret spatial relationships involving the quantitized data. For instance, researchers might analyze the concentration of certain themes or subjects within interview responses or survey responses to one or more open-ended items by location, or might examine how community sentiments vary across different urban or rural areas.

As an illustration, Onwuegbuzie et al. (2020) investigated challenges experienced by 1,932 students at Stellenbosch University that hindered their ability successfully to learn online during the emergency remote teaching that began in April 2020 due to the COVID-19 pandemic via an online questionnaire that they completed. The researchers' analysis of their responses to an open-ended question led to the identification of seven (challenge) themes that categorized the students' challenges during the COVID-19 pandemic. As was the case for Onwuegbuzie et al.'s (2007) study, each of the seven themes was quantitized by assigning a “1” to participants whose responses represented that theme, and a “0” to all others. This quantitizing led to what Onwuegbuzie (2003) referred to as an inter-respondent matrix of themes that consisted of “1s” and “0s.” Subsequently, each theme was mapped across the nine provinces of South Africa, revealing insightful patterns. For instance, the GIS map generated from these quantitized data showed that a significantly higher proportion of students experiencing issues categorized under the Internet Connection theme (i.e., that characterized students' challenges related to Internet connectivity) resided and worked remotely in Eastern Cape—the poorest province—compared to other provinces.

Spatial-based quantitizing thus bridges the gap between qualitative richness and quantitative precision, enabling researchers to harness the power of spatial analysis in understanding complex datasets where location is a key factor.

###### Time-based quantitizing

Time-based quantitizing refers to the process of converting qualitative data into quantitative forms that emphasize temporal aspects. This approach is particularly relevant in studies wherein the timing, sequence, or duration of events is crucial to understanding the dynamics and developments within the data. Time-based quantitizing helps researchers to measure and to analyze changes over time, to capture patterns of behavior, and to understand the sequence of events or phenomena in a structured, numerical format. It involves integrating time-related information into the quantitizing process. This could mean quantifying the length of time an event takes, the sequence in which multiple events occur, or the intervals between events. Such integration can provide insights into trends, cycles, or durations that are important for the study. Techniques such as time series analysis can be used to forecast future events based on past data, to analyze seasonal variations, or to detect trends and cycles. For studies focused on the occurrence and timing of events, event history analysis or survival analysis can be used. This method deals with the timing of specific events (e.g., failure times) and can accommodate data that are right-censored (i.e., when an observation ends without the event occurring). In psychological research, time-based quantitizing might be used to study the progression of a psychological condition over time. By quanitizing the frequency and intensity of symptoms reported in qualitative interviews at regular intervals, researchers can apply statistical methods to analyze changes in symptom patterns, to correlate them with treatment interventions, or to predict future outcomes.

Returning to Onwuegbuzie et al.'s (2020) study, although the researchers did not examine time-based data, they could have done so by asking the same open-ended question (i.e., What challenges are you experiencing that are hindering your ability to learn online during the COVID-19 pandemic?) at multiple time points during the pandemic. The themes that emerged at each time point then could have been quantitized (e.g., using the dichotomization process [i.e., “1” vs. “0”] described earlier) and then consolidated into a single inter-respondent matrix. This consolidated inter-respondent matrix then could have been used to conduct time-based analyses that included the following:

- *Logistic Regression for Repeated Measures*: This method identifies predictors of thematic prevalence, carefully accounting for the complex correlations among repeated observations from the same participant.
- *Survival Analysis*: Used to analyze time-to-event data, along with its discrete-time counterpart, which assesses the probability of theme emergence within specific intervals.
- *Generalized Estimating Equations*: This approach uncovers the evolving impact of predictors over time, providing insights into their dynamic effects.
- *Generalized Linear Mixed Models* (*GLMM*): These models accommodate the complexity of variations between participants, effectively managing diverse individual data points.
- *Mixed Models for Binary Outcomes*: This technique examines themes over time, capturing both fixed and random effects to delineate trends at the population level as well as individual differences.

In summary, time-based quantitizing transforms rich, qualitative temporal data into structured quantitative formats, making it possible to apply statistical analysis tools to understand complex temporal dynamics and relationships within the data. This approach is essential for any research wherein time is a pivotal factor in understanding the phenomena under investigation.

#### Level 3 quantitizing

Level 3 quantitizing, or third-level quantitizing, comprises cross-sectional-based quantitizing and longitudinal-based quantitizing. Each of these types of third-level quantitizing will be described in the following sections.

##### Cross-sectional-based quantitizing

Cross-sectional-based quantitizing involves conducting a cross-sectional study wherein data initially are collected or observed in a qualitative format (e.g., interviews, focus group discussion, observations, open-ended survey responses). Then, these qualitative data are quantitized. These newly quantitized data then are analyzed to draw conclusions about the prevalence, associations, or distributions of certain variables within the population at that specific point in time.

This approach allows researchers to utilize the strengths of both qualitative and quantitative methodologies to collect rich, contextual data about a population and then to apply statistical techniques to analyze trends or patterns at a particular moment. The result can provide a broad, statistically informed snapshot of the research topic, enriched by the depth and detail typically associated with qualitative research.

##### Longitudinal-based quantitizing

Longitudinal-based quantitizing refers to the process wherein qualitative data collected in a longitudinal study are systematically converted into quantitative data. This allows researchers to utilize qualitative methods to collect rich, detailed qualitative data at multiple time points. Quantitizing techniques then are applied to transform these data into a format that can be quantitatively analyzed. Statistical methods are employed to examine how coded categories change over time, to identify trends, and potentially to infer causal relationships. This approach enables researchers to leverage the depth of qualitative data along with the analytical power of quantitative methods to track and to analyze changes over time in a structured, numerically robust way. For example, a study might involve collecting annual interview data from participants about their health behaviors, followed by coding responses into quantitized data that tracks shifts in health attitudes and practices across the study period.

In essence, longitudinal-based quantitizing is a methodological strategy that marries the detailed, context-rich insights of qualitative research with the temporal analysis capabilities of longitudinal quantitative research, offering a potent tool for understanding how phenomena evolve over time. This integrative approach not only enhances the depth and clarity of data interpretation but also facilitates the examination of dynamic changes and trends within complex datasets, thereby allowing researchers to draw more nuanced and informed conclusions about the underlying processes driving these changes.

#### Level 4 quantitizing

Level 4 quantitizing, or fourth-level quantitizing, comprises retrospective quantitizing and prospective quantitizing. Each of these types of fourth-level quantitizing will be described in the following sections.

##### Retrospective quantitizing

In retrospective quantitizing, the initial qualitative data might have been collected for other purposes or in a naturalistic setting without a priori intentions of quantitative analysis. For example, this could involve clinical notes, interview transcripts, or open-ended survey responses collected in past studies. Retrospective quantitizing then would involve applying quantitizing techniques to these existing qualitative data; researchers can retrospectively code and quantify the data. This might include categorizing textual responses into numerically analyzable data or scoring narrative descriptions based on intensity, frequency, or presence of certain themes. The quantitized data then can be analyzed to uncover trends, patterns, or relationships that were not originally quantitized during the initial data collection phase. This approach allows researchers to utilize existing qualitative data to perform new quantitative analyses without the need to conduct fresh data collection, which can be particularly useful in contexts wherein prospective data collection is challenging, costly, or impractical. It provides an opportunity retrospectively to analyze and potentially to uncover new insights from past qualitative data using quantitizing techniques, expanding the utility and value of previously collected data.

**Figure 4:**
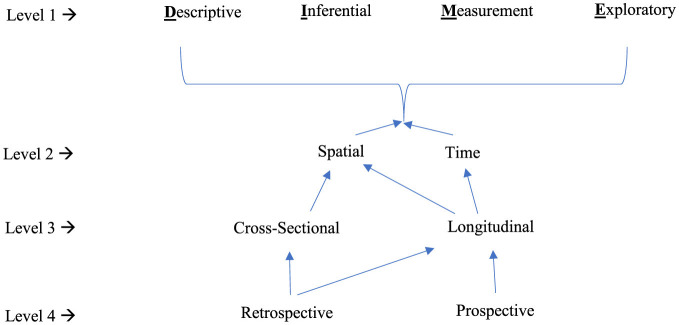
DIME-driven model of quantitizing.

In Figure 4, it can be seen that an arrow goes from retrospective quantitizing to both cross-sectional-based quantitizing and longitudinal-based quantitizing, which indicates that retrospective quantitizing can comprise both cross-sectional-based quantitizing and longitudinal-based quantitizing. This dual categorization of retrospective quantitizing is explained by understanding that the term “retrospective,” which indicates that the study data already exist and, thus, can be extracted for quantitizing. Retrospective quantitizing can involve cross-sectional quantitizing if it involves quantitizing existing data at one point in time. Also, it can involve longitudinal quantitizing if it involves quantitizing existing data across multiple time points to observe changes over time.

Consistent with this conceptualization of retrospective quantitizing, in the context of research designs, for example, Andrade (2022) explains that research designs simultaneously can describe studies as being both retrospective and cross-sectional, or retrospective and longitudinal. This dual categorization depends on how the existing data are utilized—either as a single snapshot (i.e., cross-sectional) or over multiple time points (i.e., longitudinal; Andrade, 2022).

##### Prospective quantitizing

In prospective quantitizing, researchers start with a clear plan to collect qualitative data that they intend to convert into quantitative data. This plan would be integral to the research design and involve predetermined methods for how qualitative data (e.g., interview transcripts, observations, open-ended survey responses) will be systematically coded into quantitative formats. Data would be collected prospectively, meaning that researchers would collect qualitative information as events unfold or as they interact with study participants over time. This ongoing collection could be structured in a way to facilitate subsequent quantitizing, such as using semi-structured interview techniques that align with a pre-defined coding scheme.

Once the data have been collected, the qualitative information would be converted into quantitative form according to the initial plan. These quantitized data then could be used to perform statistical analysis to identify trends, patterns, and possibly causal relationships, providing robust insights into the dynamics of the study's focus. This prospective quantitizing allows researchers to blend the depth and richness of qualitative data with the rigor and scalability of quantitative analysis, planned from the outset of the study. This prospective approach to quantitizing can enhance the precision and applicability of research findings, especially in fields wherein both contextual and measured data are crucial for understanding complex phenomena.

### Conclusions regarding the DIME-driven model of quantitizing

The DIME-driven model of quantitizing is hierarchical, wherein each subsequent level incorporates and builds on the methods and insights of the preceding levels. Specifically, Level 4 subsumes Level 3 as a special case, which, in turn, subsumes Level 2, and Level 2 subsumes Level 1. This hierarchical structure ensures that each level adds complexity and depth to the quantitizing process, providing a comprehensive framework that moves from basic descriptive quantitizing to more complex analytical tasks involving spatial, temporal, and prospective/retrospective analyses.

Overall, as can be seen, each level in the DIME-Driven Model progressively builds on the previous one, incorporating additional dimensions of analysis (spatial, temporal, cross-sectional, and longitudinal) and increasing the complexity and sophistication required for quantitizing qualitative data. This structured approach allows researchers to select the appropriate level of analysis based on their research questions and the nature of their data, enhancing the depth and rigor of their quantitative analysis. Specifically, Level 2 involves complex spatial analysis techniques and the integration of geographic data, adding a spatial context to the quantitizing process, as well as adds the challenge of accounting for temporal dependencies and changes over time, requiring advanced statistical models to analyze trends and patterns. Level 3 requires analysis techniques to control for cross-sectional differences and to ensure that comparisons are valid across different groups, as well as adding complexity through the need to handle longitudinal data structures, requiring techniques that can account for repeated measures and temporal dynamics. Finally, Level 4 involves complex historical data analysis, requiring techniques accurately to interpret and to quantitize past events and conditions, as well as adding complexity through the need for designing studies that accurately can capture and quantitize future data, often involving predictive modeling and forward-looking analysis techniques.

This four-level model of quantitizing not only systematizes the process of transforming qualitative data into quantitative analysis but also enhances the capability to handle complex data sets and to derive meaningful interpretations that are statistically robust and empirically informative. By methodically escalating through increasingly sophisticated levels of data analysis, the model facilitates a deeper integration of qualitative nuances within a quantitative framework. This progression enables researchers to uncover hidden patterns, to validate theoretical constructs, and to extend their findings to broader applications with greater confidence and precision. Ultimately, this comprehensive approach enriches scientific inquiry in general and mixed methods research in particular by bridging the qualitative-quantitative divide with rigor and creativity.

This DIME-driven model is a paradigm shift, elevating the practice of quantitizing to a realm of heightened sophistication and multidimensional analysis within the sphere of mixed methods research. By embracing this hierarchical model, scholars and researchers are afforded a beacon that navigates the often-nebulous terrain of data transformation, providing clear milestones and checkpoints along the scholarly expedition of mixed methods research. This model not only pioneers new frontiers in methodological rigor but also embodies a visionary standard for future explorations in the realm of data integration.

### *Who* should quantitize?

Quantitizing holds broad appeal due to its versatility across various research methodologies, making it a valuable tool not only for mixed methods researchers but also for those primarily engaged in either qualitative research or quantitative research. Mixed methods researchers, for instance, find quantitizing particularly useful as a means to enrich and to expand on their quantitative findings, seamlessly integrating this technique into the latter stages of their studies.

Similarly, qualitative researchers stand to gain from adopting quantitizing techniques to structure their data more systematically. This approach allows them to enhance the rigor of their analyses without necessarily transforming their studies into mixed methods research. By incorporating quantitizing within their existing qualitative frameworks, they can deepen their analytical capabilities and derive quantifiable insights from their data collections.

Quantitative researchers also benefit from the application of quantitizing techniques, especially when they engage in methods that necessitate the analysis of qualitative data. An example of such an approach is qualitative comparative analysis, which involves a systematic examination of similarities and differences across cases (De Block and Vis, 2019). This method serves as a powerful theory-building tool, enabling researchers to make connections among established categories and to refine and to test these categories further. Thus, quantitizing not only bridges the gap between qualitative and quantitative domains but also enhances the depth and scope of research across the spectrum.

#### The role of research philosophy in the process of quantitizing

In order to conduct a nuanced analysis of whether researchers representing major research philosophies should engage with quantitizing, it is crucial to consider how well the fundamental principles of these philosophies align with the procedures and implications of quantitizing. This approach involves a thorough examination across the spectrum of research traditions, encompassing qualitative, quantitative, and mixed methods-based philosophies. Such an examination is essential to determine the degree of compatibility between each philosophy's core assumptions and stances and the quantitizing process.

Tables 1–3 in the subsequent discussion provide a detailed exposition of this analysis. Each table systematically outlines the assumptions and stances of the most prevalent research philosophies within the qualitative, quantitative, and mixed methods research domains, respectively. Additionally, these tables assess the propensity of each philosophy to embrace quantitizing. This assessment is based on how effectively quantitizing can integrate with the epistemological, ontological, axiological, and methodological components of these philosophies without compromising their integrity and objectives. By exploring these dimensions, these tables provide a comprehensive overview, thereby facilitating a deeper understanding of which researchers can appropriately and effectively apply the process of quantitizing within the landscape of research methodologies.^4^

**Table 1:** Most common qualitative-based research philosophies: assumptions and stances and the likelihood of embracing the quantitizing process.

**Research philosophy**	**Ontology**	**Epistemology**	**Axiology**	**Methodology**	**Likelihood of quantitizing**	**Rationale**
Social constructionism (Berger and Luckmann, 1967; Leeds-Hurwitz, 2009; Lock, 2010; Schwandt, 2000)	Reality is socially constructed.	Knowledge is created within social contexts.	Values are openly acknowledged and scrutinized.	Common approaches include discourse analysis, ethnographic studies, and narrative research.	Generally skeptical	Perceived as reducing the richness of social phenomena.
Social constructivism (Palinscar, 1998; Rust et al., 2005; Vygotsky, 1962, 1978)	Reality is constructed through human mental activity and social interactions.	Knowledge is constructed individually but mediated socially.	Values the subjective experience and perspective of the researcher and participants.	Prefers qualitative approaches such as interviews and case studies.	Usually limited	Prioritizing individual meaning-making which is difficult to quantify. Focusing on depth over breadth in data.
Radical constructivism (Glasersfeld, 1995; Steffe and Thompson, 2000)	Knowledge and reality are personally constructed, not discoverable; rejecting an objective reality.	Understanding is subjective, shaped by experiences.	Highly reflexive about the influences of the researcher's biases; Highly individualized perspective on values.	Emphasizes qualitative approaches that explore individual experiences; Introspective and reflective qualitative methods.	Rarely embraced	It contradicts the emphasis on individual subjective experiences.
Critical theory (Habermas, 1984; Tyson, 2023)	Social reality is shaped by power, economics, and social forces.	Knowledge is a social product, influenced by material conditions and power dynamics. Knowledge is a tool for emancipation and critique.	Committed to emancipation and challenging status quo, as well as to social justice and change.	Qualitative approaches aimed at uncovering power structures and exploring individual experiences.	Open to quantitizing	If it serves critical insights and emancipatory goals.
Feminist standpoint theory (Cabrera et al., 2020)	Reality is seen through the lens of women's experiences.	Knowledge is situated; marginalized perspectives offer a more complete view of reality. Knowledge is derived from the lived experiences of marginalized groups.	Values the experiences and voices of the marginalized. Advocacy and empowerment are central.	Qualitative, focusing on the experiences of women and other marginalized groups; often narrative and ethnographic.	Limited use	If it supports advocacy goals.
Feminist theory (Disch and Hawkesworth, 2016; hooks, 2000)	Social reality is gendered and constructed through power relations. Gendered perspectives are central to understanding reality.	Emphasizes the importance of gender as a category of analysis. Knowledge is influenced by gender power relations.	Committed to revealing and challenging gender inequalities.	Qualitative, incorporating diverse women's voices.	Can be embraced	If it supports feminist objectives and serves to highlight inequalities.
Critical race theory (Delgado and Stefancic, 2023; Ladson-Billings and Tate, 1995; Lynn and Dixson, 2021)	Holds that racism is an ingrained feature of society, structurally embedded within systems and institutions.	Emphasizes the validity of experiential knowledge from marginalized groups, using narratives and storytelling to uncover racial injustices.	Values social justice and aims to dismantle racial hierarchies.	Primarily employs qualitative methods like storytelling, counter-storytelling, and analysis of cultural artifacts.	Limited use	Cautious use of quantitizing because it might reduce complex social issues to numerical data, potentially obscuring the depth of racial issues. Primarily used to support or to highlight qualitative findings.
Postmodernism (Jameson, 2014; Taylor and Winquist, 2002)	Questions the stability of the social world and denies a single, unifying reality. Skeptical of grand narratives; reality is fragmented and pluralistic.	Knowledge is contingent, fragmented, historically situated, constructed, and varied.	Often rejects grand narratives; values plurality and diversity of interpretations. Challenges meta-narratives and totalizing explanations.	Prefers qualitative methods that emphasize complexity and contradiction; Diverse, often deconstructive or ironic.	Typically resists quantitizing	Instead, valuing multiplicity and the deconstruction of categories. Generally opposed due to its challenge to singular truths.
Post structuralism (Dillet, 2017; Williams, 2022)	Reality is constructed through discourse; structures are both enabling and constraining. Structures and human relationships are fluid, not fixed.	Knowledge is produced within specific historical and cultural contexts. Knowledge is constructed through discourses and language.	Critically examines how power affects knowledge production.	Uses textual analysis, discourse analysis and other qualitative methods.	Seldom of interest	Focus is on deconstruction of texts and discourses and on how narratives and knowledge are constructed.
Symbolic interactionism (Blumer, 1969; Carter and Fuller, 2015)	Reality is constructed through social interactions and the use of symbols.	Knowledge is constructed through social interactions. Knowledge comes from the interpretation of these interactions.	Emphasizes understanding the subjective meanings and symbols individuals use. Often implicit, focusing on subjective meanings.	Uses qualitative methods such as observational studies and grounded theory; interpretative qualitative methods.	Occasionally of use.	When the goal is to generalize findings.
Phenomenology: (Moustakas, 1994) At least 29 phenomenological-based philosophies: Descriptive phenomenology (Giorgi, 2009; Todres and Holloway, 2004) Interpretive phenomenology (Tuohy et al., 2013) Reflective/Transcendental phenomenology (Husserl, 1970) Dialogical phenomenology (Herman, 2007) Empirical phenomenology (Mortari et al., 2023) Existential phenomenology (von Eckartsberg, 1998) Hermeneutic phenomenology (Laverty, 2003) Social phenomenology (Chelstrom, 2012) Psychological phenomenology (Giorgi, 1985) Ethnographic phenomenology (Rodgers, 2021) Genetic phenomenology (Lohmar, 2011) Constitutive phenomenology (Sandmeyer, 2008) Narrative phenomenology (Maggio, 2016; Shibolet, 2019; Zafran, 2020) Ethical phenomenology (Kirchin, 2003) Ecological phenomenology (Kule, 2018; van der Schyff, 2010; Wood, 2001) Neuro-phenomenology (Featherstone et al., 2013; Peters, 2000) Eidetic phenomenology (Purcell, 2010) Post-intentional phenomenology (Soule and Freeman, 2019; Vagle and Hofsess, 2016) Transpersonal phenomenology (Laughlin and Rock, 2021; Levin, 1988) Intercultural phenomenology (Hong, 2023) Phenomenological anthropology (Schnegg, 2023) Phenomenological sociology (Ferguson, 2006; Overgaard and Zahavi, 2009; Srubar, 1984) Aesthetic phenomenology (Vandenabeele, 2016) Phenomenology of perception (Merleau-Ponty, 2013) Phenomenology of religion (Cox, 2010) Feminist phenomenology (Oksala, 2004) Phenomenology of time (Kortooms, 2002) Political phenomenology (Bedorf and Herrmann, 2019) Phenomenology of embodiment (Moran, 2013)	In general, the focus is on the lived experience and essence of phenomena.	Knowledge is subjective and grounded in individual experience.	In general, the emphasis is on the importance of the researcher's openness to participants' experiences.	Uses qualitative methods such as in-depth interviews and participant observation.	Rarely used, except for empirical phenomenology (which relies on observations and descriptions that can be quantified; quantitizing can enhance the breadth and generalizability of findings), neuro-phenomenology (which involves linking phenomenological accounts with neurological data, often requiring quantitative measures to correlate experiential data with brain activity), eco-phenomenology (to understand better ecological data and patterns that often require quantitative analysis for environmental studies), psychological phenomenology (can incorporate quantitizing to explore broader psychological trends and patterns, potentially employing statistical methods to generalize findings)	Most phenomenological approaches and ethnomethodology prioritize qualitative methodologies due to their focus on deep, contextual, and interpretative aspects of human experiences. Quantitizing is generally less common and often viewed as potentially detracting from the depth and integrity of the phenomenological inquiry—especially for hermeneutic phenomenology (focused on deep interpretation of texts and experiences, it relies heavily on qualitative analysis to uncover meanings within historical and cultural contexts), existential phenomenology (centers on individual existence and personal experiences, often exploring profound existential themes that are difficult to quantify), transpersonal phenomenology (explores dimensions of human experience that transcend the individual, often delving into spiritual or transcendent aspects that are not easily quantified), reflective/transcendental phenomenology (focus on subjective introspection and essence often leads to a rejection of quantitizing, which is seen as potentially oversimplifying or misrepresenting the depth of lived experiences), dialogical phenomenology (emphasizes understanding through dialogue and relational interactions, wherein the richness of conversational context is more meaningful than quantitative data), ethical phenomenology (centers on moral and ethical dimensions of experiences, which are intrinsically qualitative and subjective, making quantitizing less relevant and rarely embraced), anti-conflationist phenomenology (emphasizes the separation of methodologies to maintain epistemological purity, likely rejecting quantitizing as it could blur the clear methodological distinctions valued by this approach)

Adapted from “Philosophical assumptions and stances of the most common mixed methods research-based research philosophies,” by Onwuegbuzie, 2024. Dialectical Publishing, pp. 10–12. Copyright 2024 by Dialectical Publishing.

#### Qualitative research-based philosophies

Table 1 illustrates the propensity of each qualitative research philosophy to embrace quantitizing, as influenced by its epistemological, ontological, axiological, and methodological underpinnings. The assessments in this table reflect the assumptions and stances of each philosophy on understanding human experience and social reality. This table reveals that researchers who value empirical, generalizable data for advocacy or broader social analysis are more likely to incorporate quantitizing readily. Conversely, those deeply committed to subjective, individual, and context-specific understandings tend to reject quantitizing—or, at least, are less likely to incorporate it—viewing it as fundamentally incompatible with their epistemological and methodological approaches.

Consistent with this observation, the qualitative philosophies whose proponents are most likely to embrace quantitizing appear to be critical theory and feminist theory. Critical theory is compatible with quantitizing when it serves to expose social inequalities or power imbalances. As such, critical theorists may quantitize to bolster arguments for social change, making this approach somewhat more amenable to integrating quantitizing when it serves their emancipatory goals. Similarly, feminist theory is compatible, and feminist theorists may quantitize effectively to highlight and to challenge gender inequalities. Quantitizing data can be used to demonstrate systemic issues, such as pay gaps or statistical discrepancies in healthcare, which align with feminist goals.

In contrast, the qualitative research-based philosophy whose proponents are least likely to embrace quantitizing appears to be symbolic interactionism. Although not completely opposed to quantitizing, symbolic interactionism places a strong emphasis on understanding individual and group interactions at a detailed, interpretive level. Quantitizing techniques are less common but might be used to some extent to generalize findings from qualitative analyses. Even more opposed to quantitizing are the qualitative philosophies of postmodernism, poststructuralism, and phenomenology in many of its various forms. Proponents of postmodernism and poststructuralism are highly skeptical of grand narratives (i.e., metanarratives) and the reduction of complex social phenomena to simple explanations or categorizations. These perspectives fundamentally critique the structures and power dynamics embedded within traditional methods of knowledge production, including quantification, which they view as an attempt to impose order and linearity on inherently disorderly and fragmented realities. Phenomenology, in general, aims to delve deeply into the lived experiences and perceptions of individuals, seeking to capture the essence of these experiences without dilution or abstraction. Quantitizing, by its nature, abstracts and generalizes individual experiences into numerical data, which opposes the phenomenological commitment to capturing the depth and nuance of direct personal experiences. Nevertheless, aside from these exceptions, the vast majority of qualitative research philosophies do not prevent researchers operating under them from engaging in quantitizing when it assists in addressing the underlying research question(s).

#### Quantitative research-based philosophies

Table 2 illustrates the propensity of each quantitative research philosophy to embrace quantitizing, as influenced by its epistemological, ontological, axiological, and methodological underpinnings. This table shows that each philosophy's approach to quantitizing qualitative findings varies, primarily based on its ontological commitments and epistemological approaches. Philosophies that prioritize objectivity and empirical verification are more likely to embrace quantitizing. In contrast, those that value subjective human constructs or mental processes are generally less inclined to do so. Additionally, the acceptance or rejection of quantitizing by proponents of these philosophies also depends on their perspectives on the nature of mathematical entities, the origins of mathematical knowledge, and their values concerning mathematics and knowledge acquisition.

**Table 2:** Most common quantitative-based research philosophies: assumptions and stances and the likelihood of embracing the quantitizing process.

**Research philosophy**	**Ontology**	**Epistemology**	**Axiology**	**Methodology**	**Likelihood of quantitizing**	**Rationale**
Platonism (Mathematical Realism; Balaguer, 1998; Tomšič, 2017)	Abstract mathematical entities exist independently of human thought; timeless, non-physical objects.	Knowledge is attained through intellectual intuition and logical reasoning.	Values the discovery of absolute and universal truths.	Deductive reasoning and discovery of eternal truths.	High	Quantitizing complements the search for universal mathematical truths.
Nominalism (Mathematical Constructivism; Kerkhove and Van Bendegem, 2012; Szabo, 2003)	Denies the independent existence of mathematical entities; sees them as constructs or labels.	Knowledge is a human construct, dependent on social practices and language.	Values practical utility and coherence.	Constructive mathematics and verification through consistency within mathematical systems.	Low	Prioritizes human-centric constructs over objective quantification.
Structuralism (Sturrock, 2008)	Emphasizes the structures or relationships among mathematical entities, rather than the entities themselves.	Knowledge arises from understanding these structures.	Values insight into the structural aspects of mathematics.	Analysis of relationships and patterns within mathematical systems.	Moderate	Useful for illuminating structural relationships.
Intuitionism (Dummett, 2000)	Mathematics is a mental construct, not reflecting any external reality.	Knowledge is subjective, accessed through mental processes and intuition.	Values the certainty and constructiveness of mathematical proofs.	Constructive proofs, emphasizing processes that can be intellectually grasped.	Low	Due to the focus on mental constructions rather than empirical data.
Formalism (Detlefsen, 2007)	Mathematics is about manipulating symbols according to agreed rules; the symbols don't necessarily represent real objects.	Knowledge is based on mastering these formal systems and operations.	Values logical consistency and rigor in formal systems.	Development and exploration of formal systems, independent of their interpretation.	High	Aligns well with the manipulation of formal systems and precise measurements.
Logicism (Demopoulos, 2013)	Mathematics can be reduced to logical foundations.	Mathematical truths are derived from logical truths.	Values the clarity and undeniable truth provided by logic.	Reducing mathematics to logic to prove mathematical truths.	High	Quantitizing supports the objective and universal nature of logical structures.
Empiricism (Meyers, 2006)	Mathematical knowledge is derived from experience and is empirical.	Knowledge is provisional and empirically tested.	Values empirical verification and practical applications.	Empirical observation and experimentation.	High	Crucial for linking mathematics to empirical observations.
Finitism (Ye, 2011)	Only finite mathematical constructs exist; rejects the existence of actual infinity.	Knowledge is about finite procedures and their results.	Values computability and concrete results.	Restricts mathematical practice to finite operations.	Moderate	If the focus remains on finite and tangible outcomes.
Realism (House, 1991)	Mathematical entities exist independently of human knowledge or perception.	Knowledge of these entities is discovered, not invented.	Values the discovery of objective, independent truths.	Objective investigation and logical analysis.	High	It aids in the objective analysis and understanding of mathematical entities.
Anti-Realism (Brock and Mares, 2006; Chalmers, 2009)	Denies the objective existence of mathematical entities outside of human conceptual schemes.	Mathematical truths are dependent on human practices or conceptual frameworks.	Values the practical and explanatory power of mathematics.	Focuses on the usefulness and practical application of mathematical concepts.	Low to moderate	Depending more on its practical utility than on seeking objective truths.
Fictionalism (Fine, 1993; Suárez, 2008)	Mathematical entities are akin to fictional characters; they do not exist.	Mathematical truths are “pretended” for their utility in explaining and predicting phenomena.	Values the usefulness and explanatory power of mathematical constructs.	Utilitarian use of mathematics as a tool for explanation and prediction.	Moderate	Appreciated for its practical benefits rather than its truth.
Psychologism (Crane, 2014)	Mathematics is a product of human thought and psychological processes.	Mathematical knowledge is derived from and limited by human cognitive capacities.	Values the understanding of human cognitive processes in mathematics.	Psychological investigation into how mathematical thoughts and processes develop.	Low	Focuses more on qualitative insights into human cognition.
Objectivism (Peikoff, 1993)	Reality exists independently of consciousness; specific principles govern reality, including mathematical ones.	Knowledge is based on objective observation and rational integration.	Rational inquiry and empirical evidence.	Rational inquiry and empirical evidence.	High	It aligns with the pursuit of objective knowledge through rational and empirical means.
Postpositivism (Phillips and Burbules, 2000; Popper, 2002)	Acknowledges that scientific knowledge is imperfect and theory-laden.	Knowledge is provisional and subject to revision; emphasizes critical testing of theories.	Values rigorous testing, critical thinking, and acknowledges the fallibility of scientific inquiry.	Scientific methods with a recognition of their limitations; uses qualitative and quantitative research.	High	But with a critical stance, recognizing the limits and potential biases of quantitative methods.

Adapted from “Philosophical assumptions and stances of the most common mixed methods research-based research philosophies,” by Onwuegbuzie, 2024. Dialectical Publishing, p. 13. Copyright 2024 by Dialectical Publishing.

Consistent with this observation, the quantitative research philosophies whose proponents are most likely to embrace quantitizing appear to be Platonism (i.e., mathematical realism), formalism, logicism, objectivism, empiricism, and postpositivism. Advocates of Platonism strongly embrace quantitizing because it aligns with the discovery of eternal, objective, immutable mathematical entities that are thought to exist independently of human thought. Proponents of formalism highly value precise and systematic manipulation of formal systems and symbols, aligning closely with the goals and methods of quantitizing. Logicism, because it reduces mathematics to logical structures, it inherently values the clarity and rigor that quantitizing can provide, wherein quantitizing supports logical deduction and the establishment of universal truths. Objectivists, with their strong emphasis on rationality and empirical evidence, highly value quantitizing as tools for discovering and verifying objective reality, as well as for validating mathematical and scientific theories. Empiricists strongly support quantitizing because of its basis in empirical verification of theories through observable phenomena, which often involves quantifying data. Postpositivists value empirical verification and scientific methodologies, making them highly supportive of quantitizing as a means to validate and to refine theories against observable data. Proponents of these philosophies who highly embrace quantitizing typically value objective, measurable, and empirically verifiable data. They appreciate the precision, clarity, and empirical grounding that quantitizing provides. In fact, they regard quantitizing as an integral part of exploring, understanding, and validating mathematical entities and theories.

In contrast, the quantitative research philosophies whose proponents are least likely to embrace quantitizing appear to be intuitionism, psychologism, fictionalism, nominalism (mathematical constructivism), and anti-realism. Proponents of intuitionism place emphasis on personal, intuitive understanding rather than on external validation methods such as quantitizing. They view mathematics as a mental construct and place significant value on intuitive understanding and mental constructions rather than over empirical or external validation (e.g., measured or derived from physical reality). They might view quantitizing as an unnecessary or misleading step if it implies the existence of an external mathematical reality. Instead, they focus on constructive proofs and the mental processes of mathematicians. Advocates of psychologism focus on understanding mathematical knowledge as a product of human thought and cognitive processes, likely preferring qualitative insights into cognitive functions and how mathematical thinking arises over quantitizing. They might view quantitizing as being too detached from the psychological processes that it deems essential for understanding mathematical concepts. Moreover, they might reject quantitizing if it is perceived as attempting to objectify what are essentially subjective cognitive constructs. Proponents of fictionalism, although they use mathematics as a practical tool, do not necessarily see quantitative analysis as reflecting any deeper truth or reality, which might make it less reliant on quantitizing. They consider mathematical entities as fictional and primarily useful for their explanatory power. Advocates of nominalism could potentially reject quantitizing because they view mathematical entities as social constructs rather than as objective entities that can be quantitized. The focus is more on the meanings and uses of mathematical terms within human contexts. Depending on the specific flavor of anti-realism, anti-realists could reject the notion that quantitizing reveals any 'true' knowledge about mathematical entities, focusing instead on the usefulness, coherence, and practical applications of mathematical theories rather than on quantitative analysis.

These quantitative research-based philosophies are less likely to embrace quantitizing because they often view mathematical entities as subjective or contingent on human thought processes, not as external realities that can or should be quantitized. Their proponents often focus on the subjective, constructed, or pragmatic aspects of mathematical knowledge, viewing quantitative methods as less critical or even irrelevant. However, it is important to note that even these philosophies would not completely reject quantitizing but would prioritize qualitative approaches. In summary, philosophies rooted in logical, empirical, and scientific methodologies most strongly embrace quantitizing, whereas those centered on human psychological or constructivist perspectives are less inclined to do so, focusing more on the qualitative or theoretical aspects of mathematical thought.

#### Mixed methods research-based philosophies

Table 3 illustrates the propensity of each mixed methods research philosophy to embrace quantitizing, as influenced by its epistemological, ontological, axiological, and methodological underpinnings. As was the case for Tables 1, 2, Table 3 shows that these philosophies reflect diverse perspectives on reality, knowledge, method, and values, each shaping the approach to mixing and integrating qualitative and quantitative research methods. Quantitizing is variously embraced depending on whether it aligns with the philosophical goals, particularly how it might support or enrich the understanding and outcomes of the research. Each of these philosophies has a unique stance on the use of quantitizing alongside qualitative findings, generally shaped by their underlying principles about reality, knowledge, method, and values. Where practical outcomes, integration of diverse methods, or enhancement of theoretical understanding are valued, quantitizing tends to be more favorably embraced. Conversely, where the emphasis is on maintaining methodological purity, exploring depth of individual experiences, or focusing on subjective realities, quantitizing is less favored.

**Table 3:** Most common mixed methods-based research philosophies: assumptions and stances and the likelihood of embracing the quantitizing process.

**Research philosophy**	**Ontology**	**Epistemology**	**Axiology**	**Methodology**	**Likelihood of quantitizing**	**Rationale**
Pragmatism-of-the-middle (Johnson and Onwuegbuzie, 2004; Johnson et al., 2007)	Realist and context-sensitive.	Knowledge is gained through actions, experiences, and outcomes.	Values outcomes that are practical and applicable.	Flexible, integrates qualitative and quantitative methods.	Likely embraced	It allows for practical application and enhanced understanding.
Pragmatism-of-the-right (Putnam, 2002; Rescher, 2000)	Moderately realist, recognizing an objective reality with subjective perceptions.	Knowledge is constructed with an understanding of multiple truths.	Values objective and informed research.	Combines qualitative and quantitative to enhance understanding.	Moderately embraced	It aligns with a balanced view of reality.
Pragmatism-of-the-left (Maxcy, 2003; Rorty, 1991)	Strongly anti-realist, emphasizing multiple constructed realities.	Knowledge is socially constructed.	Values diversity and multiple viewpoints.	Prioritizes qualitative methods but is open to integrating quantitative data.	Less embraced	Focus is on depth and complexity of qualitative analysis.
Anti-conflationist (Bryman, 1992; Hammersley, 1992; Layder, 1993; Roberts, 2002)	Clear separation between objective and subjective realities.	Emphasizes distinct knowledge domains.	Values methodological clarity and purity.	Opposes mixing methods that blur epistemological boundaries.	Rarely embraced	Emphasis on maintaining clear methodological boundaries.
Critical realist (Houston, 2001; Maxwell, 2004; Maxwell and Mittapalli, 2010; McEvoy and Richards, 2003, 2006)	Acknowledges a real world that influences, but is distinct from, our knowledge of it.	Knowledge seeks to uncover real underlying structures.	Values deep understanding of social structures.	Uses methods that reveal underlying mechanisms, may mix methods.	Highly embraced	Supports the uncovering of underlying mechanisms.
Dialectical stance (Greene, 2007, 2008; Greene and Caracelli, 1997; Greene and Hall, 2010; Maxwell and Loomis, 2003; Teddlie and Johnson, 2009)	Reality is shaped by dialectical processes.	Knowledge arises from the synthesis of opposing viewpoints.	Values the integration of opposing methods and ideas.	Integrates different methodologies to synthesize new insights.	Highly embraced	Enhances the synthesis of diverse insights.
Complementary strengths (Brewer and Hunter, 1989; Morse, 2003)	Recognizes the specific strengths of qualitative and quantitative realities.	Each method provides unique and valid insights.	Values the integrity of each methodological approach.	Maintains separation of methods to preserve unique contributions.	Less embraced	Focus on preserving the unique contributions of each method.
Transformative-emancipatory (Mertens, 2003, 2007, 2010; Mertens et al., 2010)	Reality is seen through the lens of power dynamics and inequality.	Knowledge is a tool for empowerment and change.	Values research that supports social justice.	Chooses methods that support social change.	Highly embraced	If it aids in promoting social justice and transformation.
A-paradigmatic (Patton, 2002; Reichardt and Cook, 1979)	Does not adhere to any specific reality constructs.	Practical knowledge shapes method choice.	Values pragmatism and utility in outcomes.	Flexible, driven by research questions.	Highly embraced	Supports pragmatic and practical research outcomes.
Substantive theory (Chen, 2006)	Reality interwoven with theoretical frameworks.	Knowledge is deeply contextual and theoretical.	Values theoretical coherence and depth.	Theory-driven, may integrate methods based on theory needs.	Highly embraced	If it enhances theoretical understanding.
Communities of practice (Denscombe, 2008)	Socially constructed realities within professional communities.	Knowledge evolves from communal practices.	Values the practices and traditions of communities.	Adapts methods to fit community practices.	Moderately embraced	Helps in understanding and generalizing community-specific insights.
Phenomenography (Feldon and Tofel-Grehl, 2022)	Focuses on the range of possible experiences.	Knowledge is the variation in perceptions of phenomena.	Values the depth of individual perceptions.	Qualitative, focuses on describing experiences.	Rarely embraced	Emphasis on capturing the richness of individual experiences.
Dialectical pluralism (Johnson, 2012, 2017; Johnson et al., 2014; Tucker et al., 2020)	Multiple kinds of reality acknowledged.	Integrates multiple epistemological perspectives.	Values the diversity of perspectives and methods.	Promotes dialogue among different methods.	Highly embraced	Enhances integration and synthesis of diverse perspectives.
Critical dialectical pluralism (Onwuegbuzie and Frels, 2013; Onwuegbuzie et al., 2024)	Focuses on dynamic social realities influenced by power and inequality.	Knowledge as an instrument for critiquing power structures both within the research study and the population at large.	Values social transformation and empowerment.	Aims to promote social justice through participants assuming the role of participant-researchers and researchers assuming the role of research-facilitators.	Highly embraced	If it serves social justice goals.

Adapted from “Philosophical assumptions and stances of the most common mixed methods research-based research philosophies,” by Onwuegbuzie, 2024. Dialectical Publishing, p. 14. Copyright 2024 by Dialectical Publishing.

Consistent with this observation, the mixed methods research philosophies whose proponents are most likely to embrace quantitizing appear to be pragmatism-of-the-middle, critical realism, dialectical pluralism, and critical dialectical pluralism. Pragmatism-of-the-middle likely embraces quantitizing due to its practical and outcome-oriented approach. It values the integration of methods that can enhance practical applications and outcomes. Critical realists embrace quantitizing because it helps uncover deeper truths about underlying mechanisms, aligning with its goal of revealing the real structures influencing observed phenomena. Both dialectical pluralists and critical dialectical pluralists value the integration and synthesis of diverse methods and data forms, including quantitizing, to enrich the dialogue among different epistemological perspectives, with critical dialectical pluralists especially embracing quantitizing if it serves social justice goals.

Conversely, the mixed methods research philosophies whose proponents are least likely to embrace quantitizing appear to be phenomenography, complementary strengths, and anti-conflationist. Phenomenography focuses on capturing the richness of individual perceptions and experiences primarily through qualitative means. It prioritizes depth over breadth, making quantitizing less relevant and seldom embraced. Proponents of complementary strengths, although not completely rejecting quantitizing, value the independence of qualitative and quantitative research methods. They suggest maintaining a separation to preserve the integrity of each approach, thereby not favoring the mixing or integrating of data forms. Anti-conflationist advocates likely reject quantitizing because it emphasizes the importance of maintaining clear methodological boundaries between qualitative and quantitative research methods. Anti-conflationists argue against mixing methods that could blur epistemological and methodological distinctions, favoring a more principled approach whereby each method retains its purity.

However, it should be noted that proponents of these three philosophical approaches—phenomenography, complementary strengths, and anti-conflationist—are unlikely to reject outright the use of quantitizing. Rather, they are more likely to express concerns about an over-reliance on quantitizing, cautioning against its potential to overshadow the nuanced insights provided by qualitative analysis. In their view, quantitative methods should serve to augment, rather than to overshadow, the richness of qualitative data.

In summary, proponents of mixed methods research-based philosophies that embrace quantitizing generally consider this technique as enhancing the robustness, practicality, and depth of their inquiries. They are driven by objectives that benefit from integrating quantitative rigor into qualitative contexts, such as improving practical applications, uncovering deeper systemic structures, or synthesizing diverse perspectives for richer insights. Contrastingly, philosophies least embracing quantitizing often are rooted in maintaining methodological purity, valuing depth of individual experiences, or prioritizing epistemological clarity. These advocates may view quantitizing as diluting the philosophical integrity of their methods or as inappropriate given their specific research aims and contexts. Each philosophy's stance on quantitizing reflects its underlying assumptions about the nature of reality, the process of knowledge acquisition, the purpose of research, and what is valued as legitimate and important in the research process.

### Summary of propensity for quantitizing across the three research traditions

Across the diverse landscape of the qualitative, quantitative, and mixed methods research traditions—with notable exceptions such as the qualitative philosophies of postmodernism, poststructuralism, and many forms of phenomenology—quantitizing generally aligns well with the ontological, epistemological, axiological, and methodological elements of the overwhelming majority of other research philosophies. Although not universally advocated, the compatibility of quantitative analysis techniques with these principles suggests that researchers working within these frameworks can appropriately utilize quantitizing when it serves to illuminate the research question(s) at hand. Particularly, outside of these three sets of research philosophies, this alignment allows quantitizing to play a supportive role, enhancing the empirical robustness and breadth of insights into complex phenomena.

## Summary and conclusions

Building on Sandelowski et al.'s (2009) highly cited article, the present article has delved into the evolution and application of quantitizing, a process of converting qualitative data into quantitative formats, which, despite its potential, has not seen an increase in use over the years. This stagnation is attributed mainly to the dearth of methodological articles that explore and refine the approach. In response to this gap, the article introduces a comprehensive meta-framework for quantitizing qualitative data, structured around the 5W1H approach (Why, When, What, Where, How, Who), which serves as a detailed guide for researchers.

The meta-framework incorporates several existing frameworks and models, integrating both mixed methods and multiple methods research approaches to provide a structured yet flexible guide for applying quantitizing across different research contexts. The 5W1H sections of the article systematically address the key aspects of quantitizing, starting with the reasons for its use, the appropriate contexts and timing, and detailing the specific methods and approaches that can be utilized.

One of the pivotal aspects of the meta-framework is the DIME-Driven Model of Quantitizing. This model outlines four levels of quantitizing complexity: ***D***escriptive-based, **I**nferential-based, **M**easurement-based, and **E**xploratory-based, each adding depth and precision to the analysis of qualitative data. The DIME acronym not only simplifies the recall of these categories but also emphasizes the value and precision inherent in the quantitizing process. The utility of this model lies in its ability to enhance methodological robustness and data integration, providing clearer, more actionable insights that are critical in areas such as psychological research, clinical research, and policy development.

The current article ardently advocates for a judicious application of quantitizing, proposing that it be used strategically to augment qualitative analyses. This approach is recommended as a means to enhance the clarity and practical applicability of research outcomes, without detracting from the depth and richness of qualitative data. Further, this article explores the application and value of quantitizing across qualitative, quantitative, and mixed methods research traditions, demonstrating its broad relevance and transformative potential. It discusses the variable adoption of quantitizing based on differing philosophical perspectives related to ontology, epistemology, axiology, and methodology. Despite the disparities inherent in these philosophical foundations, it is noted that very few research philosophies entirely dismiss the practice of quantitizing.

The article advocates for a balanced use of quantitizing to complement qualitative analyses and to enhance research clarity and applicability without compromising the richness of narrative data. Serving as a comprehensive resource, the aim of this article has been to elucidate the complexities and benefits of quantitizing. It is hoped that it inspires researchers to incorporate this versatile technique into their analytical repertoire, thereby enriching the depth and enhancing the practical applicability of their research findings.

## Data Availability

The raw data supporting the conclusions of this article will be made available by the authors, without undue reservation.

## Footnotes

^1^The decision to use the word “quantitizing” and its variants (i.e., *quantitize, quantitized, quantitizes, quantitising, quantitise, quantitised*, and *quantitises*) in the systematic reviews conducted for the current article (i.e., *Journal of Mixed Methods Research*, Scopus) was made for two main reasons. First, by targeting the term “quantitizing” and its variants, the review ensures the inclusion of all studies that specifically address this concept, thereby eliminating false positives. This specificity is essential for collecting consistent information on how quantitizing is defined, applied, and evaluated across different works. Second, quantitizing is a key process in mixed methods research and is one of the (few) terms that has a shared meaning among mixed methods researchers. Therefore, a focused review on quantitizing facilitates better synthesis and comparison of procedures and findings across different studies, allowing researchers to identify common themes, challenges, and effective practices associated with this specific process. Consistent with this assertion, Gough et al. (2017) provide methodological guidance on conducting systematic reviews, discussing the importance of focusing on specific terms to ensure comprehensive coverage. However, it should be noted that focusing only on the term “quantitizing” and its variants likely results in false negatives, missing authors (see, for e.g., Bazeley, 2003, 2010, 2017, 2021) who describe the process using different terms (e.g., conversion, transformation). Attempting to identify all authors who discussed or applied the concept of quantitizing without using this specific term is beyond the scope of this article because it would require reviewing every single mixed methods research article published in the Scopus database over a 24-year period to determine whether each author used the concept of quantitizing despite not using the term. This issue is an example of *terminological variance* or *terminological inconsistency*, which can lead to misunderstandings, and is particularly problematic in interdisciplinary research wherein terminologies might carry different connotations in different fields. Terminological inconsistency also complicates the process of synthesizing research concepts.

^2^Further, fitting ARIMA (i.e., AutoRegressive Integrated Moving Average) models—which represent statistical models used for forecasting time series data—to the time series data in Figure 2 suggested that the best ARIMA model identified for the data is an ARIMA(0,1,1), which means there are no autoregressive terms (AR = 0)—wherein the AR part of the ARIMA model indicates how past values in the series can predict future values; one differencing operation (I = 1)—wherein I represents the number of differencing operations of raw observations needed to make the time series stationary (i.e., to stabilize the mean over time); and one moving average term (MA = 1)—wherein MA assists in smoothing out the noise in the data. This model indicates the following: (a) There is no pattern of publication frequency that repeats over the 16 years in a way that can directly predict future publication frequency; and (b) the publication frequency is somewhat random but with some predictability when considering the immediate past fluctuation.

^3^Talk can be individual (e.g., individual interviews) or group-based (e.g., paired depth interviews, focus group discussions, critical dialectical pluralist focus group discussions), face-to-face (e.g., interviews, focus group discussions) or virtual (e.g., online focus groups, chatroom discussions, listservs), synchronous (e.g., SMS, text) or asynchronous (e.g., emails), and verbal (i.e., voice of each interviewee) or nonverbal (e.g., proxemics, kinesics, paralinguistics, chronemics; cf. Onwuegbuzie and Abrams, 2021, in press). Observations can be emic-based (e.g., onsite observations) or etic-based (e.g., Geographic Information Systems), interactive (i.e., live observations) or non-interactive (i.e., past observations), first hand (e.g., by the researcher) or second hand (e.g., by someone else). Documents can be non-digital (e.g., articles, books, newspapers) or digital (e.g., blogs, tweets, Facebook, emails, chat rooms). Finally, images can be still (e.g., drawings, paintings) or moving (e.g., videos), 2-dimensional (e.g., drawings, paintings) or more than 2-dimensional (e.g., movies), non-virtual (e.g., drawings) or virtual (e.g., I-phone, I-Pad, Youtube, TikTok, Panoramio, Flickr, iMovie, Instagram).

^4^It should be acknowledged that a researcher can holding alternative understandings of reality at the same or contiguous times by making Gestalt switches. However, unless the Gestalt switching involves one or more of the three sets of research philosophies in Tables 1–3 that do not align well with the process of quantitizing (i.e., postmodernism, poststructuralism, and many forms of phenomenology), holding alternative understandings of reality at the same or contiguous times should not prevent a researcher from appropriately utilizing quantitizing when it serves to illuminate the research question(s) at hand.
